# Central nervous system effects of GLP-1 receptor agonists: A systematic review of human neuroimaging studies

**DOI:** 10.1016/j.neubiorev.2026.106990

**Published:** 2026-09-19

**Authors:** Leyla R. Brucar, Kylee B. Miller, Nora D. Volkow

**Affiliations:** National Institute on Alcohol Abuse and Alcoholism, Bethesda, MD, USA

**Keywords:** Glucagon-like peptide-1 receptor agonists, Neuroimaging, Magnetic resonance imaging, Positron emission tomography, Obesity, Diabetes, Psychiatry, Neurodegenerative disorders

## Abstract

Interest in glucagon-like peptide-1 receptor agonists (GLP-1RAs) has expanded beyond obesity and diabetes, with growing preclinical and clinical evidence supporting potential applications in psychiatric and neurodegenerative disorders. However, the central nervous system (CNS) mechanisms engaged by GLP-1RAs remain poorly characterized, limiting the capacity to optimize their therapeutic potential. This systematic review synthesized the literature examining GLP-1RA-related CNS effects, focusing on treatment-related neural responsivity, temporal dynamics, and modulators of CNS response. PubMed and Embase were systematically searched from inception to January 2026 for human studies across structural magnetic resonance imaging (MRI), task-based and resting-state functional MRI, molecular imaging, and neurophysiological approaches. Thirty-seven studies met inclusion criteria. The reviewed evidence suggests that GLP-1RAs engage distributed cortical and subcortical brain systems. Attenuation of reward-related responsivity following acute GLP-1RA administration was among the more consistent findings. Notably, evidence suggests that GLP-1RAs differentially modulate reward-related processes rather than uniformly attenuate reward function, with neural effects further varying according to metabolic status and underlying phenotype. Several studies additionally indicated that CNS effects evolve over the course of treatment, with more pronounced neural modulation during short-term treatment and diminished or altered effects following prolonged exposure. However, the current neuroimaging literature is constrained by small sample sizes and methodological heterogeneity, limiting direct comparisons across studies and underscoring the need for greater standardization. This review provides insight into the neurobiological mechanisms engaged by GLP-1RAs, identifies emerging patterns of CNS response with potential implications for mechanistically informed treatment strategies, and highlights key mechanistic considerations and priorities for future neuroimaging research.

## 1. Introduction

Glucagon-like peptide-1 receptor agonist-based pharmacotherapies (GLP-1RAs) are highly effective treatments for metabolic disorders like type II diabetes mellitus (T2DM) and obesity (Drucker, 2024). Along with their widespread clinical adoption, interest in their therapeutic potential beyond metabolic regulation has grown significantly (De Giorgi et al., 2025; Fang et al., 2025; Klausen et al., 2022a; Volkow et al., 2026; Zhao et al., 2021; Zheng et al., 2024). These medications activate the GLP-1 receptor (GLP-1R), thereby mimicking the actions of endogenous GLP-1, an incretin hormone produced both peripherally and in the central nervous system (CNS) (Nadkarni et al., 2014). Peripherally, GLP-1 is produced by enteroendocrine L-cells in the gut, where it plays a key role in metabolic regulation including glucose homeostasis, gastric emptying, appetite and satiety, predominantly through endocrine and vagal afferent signaling (Nadkarni et al., 2014; Müller et al., 2019). Centrally, GLP-1 is synthesized by preproglucagon-expressing neurons in the nucleus tractus solitarius (NTS) of the brainstem (Müller et al., 2019; Holst, 2007). NTS GLP-1 neurons function as neuromodulators, sending projections to widely distributed GLP-1Rs throughout the brain (Llewellyn-Smith et al., 2011; Alhadeff et al., 2012). GLP-1Rs are expressed not only in metabolic centers such as the hypothalamus, but also throughout the mesolimbic system, including the ventral tegmental area (VTA), nucleus accumbens (NAc), which are critical for reward- and motivational processes, and amygdala, central to affective processing (Muscogiuri et al., 2017; Dickson et al., 2012; Alvarez et al., 2005). They are also expressed in the choroid plexus and several cortical brain regions (Botfield et al., 2017; Gupta et al., 2023; Moaket et al., 2025). Recent human brain mapping studies suggest that cortical GLP-1R expression may be more extensive than that observed in rodents and non-human primates, with relatively prominent expression in frontal and parietal cortical regions, although regional heterogeneity both within the human brain and across species is evident (Gupta et al., 2023; Moaket et al., 2025; Brain Expression Cluster, 2026; Sjöstedt et al., 2020). GLP-1Rs in the brain are expressed both in neurons and glial cells (Cui et al., 2022; Diz-Chaves et al., 2022; Fu et al., 2020), where their activation has been linked to neuroprotective processes (e.g., attenuating neuroinflammation), modulation of key neurotransmitter systems (e.g., dopamine, glutamate, GABA), neural circuits supporting reward, cognitive, and affective functioning (Zhao et al., 2021; Diz-Chaves et al., 2022; Hunter and Hölscher, 2012; Zheng et al., 2025; Merkel et al., 2025), and alterations of cerebral blood flow (Chen et al., 2025; Nizari et al., 2021). Through these signaling pathways and their widespread effect on biological systems throughout the body, GLP-1RAs are well-positioned to influence the mechanisms implicated across a range of psychiatric and neurodegenerative disorders.

Indeed, preclinical, and a small yet rapidly emerging body of clinical research have demonstrated the transdiagnostic potential of GLP-1RAs beyond metabolic disease (De Giorgi et al., 2025; Tempia Valenta et al., 2024). These findings have shown the capacity of GLP-1RAs to decrease rates of alcohol and drug use (Bruns VI et al., 2024; Farokhnia and Leggio, 2026; Volkow and Xu, 2026), mitigate symptoms of anxiety and depression (Kim et al., 2020; Chen et al., 2024), reduce binge eating behaviors (Aoun et al., 2024), and improve cognitive functioning (Ballum et al., 2026). However, evidence from randomized controlled trials (RCTs) is currently very limited. The strongest support comes from trials in alcohol use disorder (AUD), which have demonstrated positive effects for second generation GLP-1 drugs (Hendershot et al., 2025; Klausen et al., 2026), consistent with preclinical findings in rodents and non-human primates (Zheng et al., 2025). Meanwhile ongoing clinical trials may soon provide the evidence needed to corroborate their therapeutic value in various brain disorders (De Giorgi et al., 2025; Fang et al., 2025; Tempia Valenta et al., 2024; Farokhnia and Leggio, 2026; Apostolopoulou and Koufakis, 2025; Patil et al., 2026). Although many RCTs focus on metabolic, anthropometric, and clinical outcomes, such as change in alcohol/substance use days, craving, or depressive symptoms (Vinberg, 2026; Fink-Jensen, 2026; Yammine, 2026), characterizing the CNS effects of GLP-1RA treatment is also an essential component to elucidating the utility of these medications across psychiatric and neurodegenerative disorders.

Neuroimaging can help address this need by enabling in vivo characterization of brain systems engaged by GLP-1RA treatment. Different imaging modalities provide distinct yet complementary approaches to assessing these CNS effects. Structural magnetic resonance imaging (sMRI) characterizes regional brain morphology, providing measures of tissue volume and cortical anatomy (Lerch et al., 2017). Functional MRI (fMRI) measures blood oxygen level-dependent (BOLD) signal fluctuations as an indirect marker of neural activity at rest and during experimental tasks (Glover, 2011). These signals can be analyzed to assess regional brain activity, evaluate functional connectivity via temporal correlations between regions, and map the organization of large-scale functional brain networks (Biswal and Uddin, 2025). Complementing MRI-based measures of brain structure and function, positron emission tomography (PET) and single-photon emission computed tomography (SPECT) use specific radiotracers to assess physiological and molecular processes, including cerebral metabolism, neurotransmitter function, and protein deposition (Lu and Yuan, 2015). Neurophysiological approaches, in turn, quantify electrophysiological activity with greater temporal resolution, capturing the rapid dynamics of these CNS responses (Mercier et al., 2022).

Collectively, these imaging tools may help clarify putative neural mechanisms of GLP-1R agonist action, evaluate their association with clinical and psychiatric outcomes, and determine whether CNS responses to GLP-1RAs can serve as markers or predictors of treatment responsivity. This is particularly relevant given the significant interindividual variability observed across GLP-1RA treatment response (Apostolopoulou and Koufakis, 2025). In fact, across randomized trials and real-world settings, up to 30% of individuals have been classified as ‘nonresponders’, failing to achieve clinically meaningful weight loss (>5% of body weight) following GLP-1RA treatment (Davies et al., 2021; Squire et al., 2025). Treatment efficacy also appears to vary according to factors such as baseline BMI, prior GLP-1RA exposure, sex, and comorbid T2DM (Apostolopoulou and Koufakis, 2025; Squire et al., 2025; Crabtree et al., 2023; Ryan et al., 2024), underscoring the need to better understand the neurobiological mechanisms underlying differential treatment responsivity.

Neuroimaging may also help elucidate the temporal dynamics of GLP-1RA-related CNS effects, including understanding how early these effects emerge and how neural responsivity evolves over the course of treatment. Improved understanding of these dynamics may help inform treatment efficacy, dosing strategies, and broader clinical decision-making aimed at maximizing therapeutic potential. To date, a growing but heterogeneous body of neuroimaging research has examined the effects of GLP-1RAs on brain function across diverse human populations and experimental contexts. However, these findings have not yet been systematically integrated.

Here, we critically evaluate the available clinical research on the CNS effects of GLP-1RA medications. Currently, only one prior review on this topic has been published, focusing specifically on the role of GLP-1 and GLP-1R mono-agonists in modulating brain functional connectivity (Au et al., 2025). In contrast, the present review synthesizes findings on CNS effects of both GLP-1R mono- and dual-agonists across a broader range of imaging modalities and paradigms than functional connectivity alone, including structural and functional MRI (sMRI; fMRI), molecular imaging (PET; SPECT), and neurophysiological approaches. Specifically, we evaluate, 1) treatment-related CNS changes across imaging modalities, 2) potential neural modulators and predictors of treatment responsivity, 3) the temporal dynamics of CNS treatment effects, and 4) key gaps and research opportunities that may inform more effective clinical implementation and optimization of GLP-1RAs for obesity, metabolic dysfunction, and beyond.

## 2. Methods

### 2.1. Search strategy and study selection

This systematic review was conducted in accordance with the 2020 Preferred Reporting Items for Systematic reviews and Meta-Analyses (PRISMA) guidelines (Page et al., 2021). The review was not prospectively registered. Literature was systematically searched for on Pub-Med/MEDLINE and Embase databases, covering the period of inception until January 25th, 2026. The titles and abstracts of the retrieved articles were independently screened for relevance by two authors (L.R.B and K.M) and resolved through discussion and consult with a third reviewer (N.D.V). The search query used for the systematic review encompassed key terms related to GLP-1R agonism (including specific medications such as liraglutide, semaglutide, tirzepatide), combined with neuroimaging and neurophysiology modalities (e.g., MRI, fMRI, PET, SPECT, EEG, MEG) and CNS-related outcomes. The full search string is provided in the Supplementary Materials. To ensure all relevant articles were included, additional literature was identified through backward citation tracking and searches on Google Scholar.

### 2.2. Eligibility Criteria

We included empirical studies in humans that assessed the CNS effects of pharmacotherapies with GLP-1R agonist activity via neuroimaging or neurophysiology following either experimental administration or observational exposure. Eligible pharmacotherapies included GLP-1R mono-agonists and dual-agonists with GLP-1R activity, such as tirzepatide, a dual GIP/GLP-1R agonist. Studies were eligible regardless of whether CNS outcomes were primary, secondary or exploratory. Study design was not restricted, with both randomized and non-randomized, controlled and uncontrolled studies eligible for inclusion. There were also no restrictions based on clinical setting, population characteristics, duration or dose of GLP-1RA exposure. Given the growing interest of GLP-1R agonist pharmacotherapies across diverse clinical contexts, this inclusion criteria were deliberately broad to provide a comprehensive overview of the available human neuroimaging and neurophysiological evidence across study designs, populations, and methodologies. We excluded studies that were: (1) focused solely on endogenous GLP-1; (2) reviews, meta-analyses, posters, abstracts, guidelines, or protocols; (3) preclinical research; (4) non-English. Comparator type was not used as an exclusion criterion; instead, relevant GLP-1RA comparisons were selectively extracted.

### 2.3. Data Extraction and Management

Two authors (L.R.B and K.M) independently conducted full text reviews of the selected articles and extracted relevant data. Data extraction included the following: study characteristics (publication year, authors, title, design); sample characteristics (diagnoses, total and imaging-specific sample sizes, age, sex/gender, BMI); intervention characteristics (GLP-1RA, comparator[s], dosage, duration of exposure); imaging parameters (modality, paradigm, feeding state at scan, scan timepoints); analytic plan (region-of-interest versus whole-brain analysis, primary comparisons, multiple comparison correction, included covariates); and outcomes (GLP-1RA-related CNS effects, behavioral and metabolic effects). Our primary outcome of interest was CNS effects associated with GLP-1RA treatment. Accordingly, comparisons between GLP-1RA versus placebo/controls or clinically relevant glycemic comparators (e.g., insulin glargine) were retained in the primary tables to isolate GLP-1RA-specific CNS effects. Comparisons involving other active pharmacologic comparators are not discussed within the text as they were beyond the scope of this review. Given substantial methodological and clinical heterogeneity of the included studies, findings are organized by neuroimaging modality and experimental paradigm. For the purposes of data extraction and synthesis, terminology describing CNS effects, responses, modulation or associated changes are used descriptively to refer to treatment-related differences in imaging measures. These terms reflect observed changes in CNS measures and do not, by themselves, establish direct central receptor engagement or a specific receptor-mediated mechanism.

### 2.4. Quality assessment and risk of bias

Quality and risk-of-bias was evaluated using the National Institute of Health (NIH) Study Quality Assessment Tools (Ma et al., 2020; National Institute of Health, 2026). As GLP-1RA-related CNS effects were our primary outcome of interest, quality assessment was conducted with specific attention to the analytic approach, pre-specification, and statistical handling of the neuroimaging component of each included study. The NIH tools served as the primary framework for quality assessment and were supplemented with neuroimaging-specific criteria evaluating correction for multiple comparisons and the reporting and incorporation of relevant covariates in statistical analyses.

Studies in which the neuroimaging outcomes were assessed under controlled intervention conditions, including randomized allocation (including parallel- or cross-over designs), secondary analysis with preserved randomized treatment allocation, and non-randomized studies including a comparator group or condition, were evaluated using the NIH Quality Assessment of Controlled Intervention Studies tool (Ma et al., 2020; National Institute of Health, 2026). When neuroimaging outcomes were assessed using single-arm pre–post comparisons without a concurrent control group, the NIH Before–After (Pre–Post) tool was used (Ma et al., 2020; National Institute of Health, 2026). Studies reporting descriptive single-case or small comparative case-series analyses were evaluated using the NIH Quality Assessment Tool for Case Series Studies (Ma et al., 2020; National Institute of Health, 2026). Quality assessment was conducted by two independent reviewers (L.R.B and K.M), with all conflicts resolved through discussion. Final quality ratings were assigned by consensus based on NIH assessment domains and additional neuroimaging-specific criteria.

## 3. Results

### 3.1. Search results

The systematic search of the relevant literature yielded 286 studies. Five additional articles were identified through backward citation tracking and a manual Google Scholar search. After removing 40 duplicates, the 251 remaining articles underwent title and abstract screening based on the predefined inclusion and exclusion criteria. Among these, 50 articles were deemed eligible for full-text review. Thirteen studies were further excluded (reviews: n = 2; incorrect outcome: n = 5; incorrect intervention/exposure: n = 6). In total, 37 studies met eligibility criteria and were included in this systematic review (Fig. 1). Detailed characteristics and key findings are summarized throughout Supplementary Tables 1-5, organized by imaging modality.

### 3.2. Quality Assessment and Risk of Bias

Overall, the included studies were of good to fair methodological quality (Supplementary Tables 6-8). Common methodological limitations were incomplete reporting of allocation concealment and blinding procedures, as well as limited neuroimaging-specific sample size justifications. Power calculations, when reported, were often based on primary clinical rather than imaging outcomes, reflecting the frequent inclusion of neuroimaging as a substudy or secondary component of larger clinical trials. Although appropriate for the primary clinical endpoint, these calculations do not necessarily ensure adequate statistical power to detect reliable neuroimaging effects. There is no fixed relationship between the sample sizes required for clinical and neuroimaging endpoints, as requirements depend on the inferential question, study design, expected effect size, and measurement variability and reliability (Mumford and Nichols, 2008; Suckling et al., 2007). For example, reliably detecting small brain-behavior associations may require samples in the thousands compared with a median sample size of approximately 25 participants reported in neuroimaging research (Marek et al., 2022). However, such estimates are not directly generalizable to within-subject or interventional imaging designs. Measurement reliability, for fMRI specifically, can also vary according to the imaging metric (e.g., functional connectivity or graph theory-based measure) and amount of data acquired per participant (e.g., number of scans, scan duration, and number of observations contributing to task contrasts) (Nee, 2019; Ooi et al., 2025). Additional neuroimaging-specific limitations identified in the quality and risk-of-bias assessment included small sample sizes, lack of reported multiple-comparison correction in a substantial proportion of studies, and incomplete reporting and statistical handling of covariates.

### 3.3. Study characteristics

The included studies varied considerably with respect to clinical population, GLP-1RA administered, exposure duration, imaging modality, experimental paradigm, and analytic approach (Fig. 2; Supplementary Tables 1-5). The use of region-of-interest (ROI) versus whole-brain analysis varied widely amongst studies, and whether scanning was done under fasted or postprandial conditions. Sample sizes were generally small, with several investigations including fewer than 15 participants per study group.

Most investigations involved individuals with obesity and/or metabolic dysfunction, including T2DM (n = 25). Neurodegenerative populations were less frequently represented, primarily Alzheimer’s disease (AD; n = 3), followed by subjective cognitive complaints/impairment (n = 2) and Parkinson’s disease (PD; n = 1). Additional studies examined psychiatric populations, including AUD (n = 1) and major depression/bipolar disorder (n = 1); individuals with endocrine disorders, such as polycystic ovary syndrome (PCOS; n = 2); individuals with primary polydipsia (n = 1); and healthy controls (n = 1).

Liraglutide (n = 17) and exenatide (n = 16) were the most frequently investigated GLP-1R mono-agonists, with fewer studies evaluating semaglutide (n = 2), dulaglutide (n = 1), and lixisenatide (n = 1; Fig. 2). Two studies evaluated the central effects of tirzepatide, a dual GIP/GLP-1R agonist (Fig. 2). Studies varied in treatment duration, categorized as acute (single dose), sub-acute (< 4-weeks), short-term (4–12 weeks), intermediate-term (12–26 weeks), or long-term (>26 weeks). Most investigations examined acute or intermediate-term exposure, with fewer studies evaluating long-term treatment effects extending beyond 26 weeks. Several studies additionally employed repeated neuroimaging assessments across placebo and GLP-1RA conditions and/or across multiple treatment timepoints. The distribution of studies across these predefined exposure duration categories is summarized in Fig. 2, with the exact GLP-1RA exposure duration and neuroimaging scan timepoints for each study reported in Supplementary Tables 1-5.

CNS effects of GLP-1RAs were investigated using a range of neuroimaging modalities, most commonly functional MRI (fMRI; n = 28), followed by molecular imaging (PET/SPECT; n = 7), structural MRI (n = 4), and intracranial EEG (n = 1; Fig. 2). Among fMRI studies, cue-reactivity paradigms were most frequently employed (n = 17), with fewer studies utilizing food anticipation/consumption paradigms, olfactory paradigms, or cognitive and reinforcement-learning tasks. Resting-state fMRI was additionally examined in six studies.

### 3.4. Functional Neuroimaging

#### 3.4.1. Task-Based fMRI: Cue Reactivity Paradigms

Seventeen studies investigated GLP-1RA-mediated effects on cue reactivity (Supplementary Table 1). Most used food cue paradigms in individuals with obesity and/or T2DM (N=15) (ten Kulve et al., 2015; Schlögl et al., 2013; van Bloemendaal et al., 2014; van Bloemendaal et al., 2015a; Eldor et al., 2016; Farr et al., 2016a; Farr et al., 2016b; Bae et al., 2019; Binda et al., 2019; Farr et al., 2019; van Ruiten et al., 2022a; van Ruiten et al., 2022b; van Ruiten et al., 2022c; Jensterle et al., 2025; Martin et al., 2025), whereas one study investigated beverage cue reactivity in individuals with primary polydipsia (Winzeler et al., 2021) and another examined alcohol-related cue responsivity in AUD (Klausen et al., 2022b). The majority of cue reactivity studies assessed CNS responses under fasted conditions, although four additionally investigated postprandial responses to determine whether GLP-1RA effects differed according to feeding state (ten Kulve et al., 2015; Farr et al., 2016a; van Ruiten et al., 2022a; Jensterle et al., 2025). Twelve studies employed ROI-based analyses, predominantly focusing on the insula, striatum, amygdala, and orbitofrontal cortex (OFC), whereas the remaining studies utilized whole-brain approaches.

Across food cue paradigms, GLP-1RA administration was most consistently associated with attenuated activation within canonical reward-related regions, including the striatum, OFC, insula, and amygdala (ten Kulve et al., 2015; van Bloemendaal et al., 2014; Eldor et al., 2016; Farr et al., 2016a; van Ruiten et al., 2022b; Jensterle et al., 2025; Martin et al., 2025). In obesity, acute GLP-1RA administration generally demonstrated reduced activation to food cues across these regions, with effects measured within 30-minutes of exenatide administration (van Bloemendaal et al., 2014; Eldor et al., 2016). Several studies additionally reported more pronounced attenuation of food cue responsivity in individuals with obesity and/or T2DM relative to lean comparators (van Bloemendaal et al., 2014; Eldor et al., 2016; Bae et al., 2019), although findings were not entirely consistent across studies (van Bloemendaal et al., 2014; Binda et al., 2019). Beyond reward-related circuitry, modulation of hypothalamic activation following acute administration was also reported, suggesting concurrent engagement of homeostatic circuitry, although the direction of these effects varied across paradigms and populations (Schlögl et al., 2013; Eldor et al., 2016; Bae et al., 2019).

Beyond acute administration, findings from sub-acute to short-term treatment (< 12 weeks) and intermediate- to long-term treatment (≥ 12 weeks) suggested more variable and often time-dependent CNS effects. In exenatide-treated individuals, reduced activation to high-calorie food cues alongside increased activation to low-calorie cues within the putamen was observed after approximately 10 days of treatment (van Ruiten et al., 2022b, 2022c). These effects, however, were no longer evident following 16 weeks, despite continued weight loss. Similar temporal patterns were observed with liraglutide: early reductions in food cue responsivity across reward-related regions were detected following sub-acute and short-term administration (ten Kulve et al., 2015; Farr et al., 2016b, 2019; Martin et al., 2025), but diminished following prolonged treatment despite continued anthropometric and metabolic improvement (ten Kulve et al., 2015; Farr et al., 2019; Martin et al., 2025). Notably, only after adjusting for weight-loss, Farr et al. (2019) observed increased OFC activation after 5 weeks of liraglutide, which the authors interpreted as a potential ‘counter--regulatory’ response during prolonged treatment. Similarly, tirzepatide reduced activation to highly desirable food cues in frontal, orbitofrontal, cingulate and hippocampal regions after 3 weeks of treatment, although these effects were no longer significant after 6 weeks despite continued behavioral and weight-related improvements (Martin et al., 2025). In contrast, semaglutide treatment in women with PCOS reduced post-prandial, but not fasted, food cue activation after 16 weeks (Jensterle et al., 2025), further suggesting that feeding state may influence GLP-1RA CNS responsivity.

In addition to reward- and homeostatic-related circuitry, two studies reported modulation of visual and sensory processing regions (Bae et al., 2019; Binda et al., 2019). Specifically, acute exenatide administration was associated with decreased activation in temporal pole and increased activation in extrastriate occipital regions, regardless of obesity status and largely independent of cue type (Binda et al., 2019), suggesting that GLP-1RAs may influence attentional or visual processing in addition to food-specific reward responsivity. Similarly, Bae et al. (2019) observed reduced activation in the fusiform gyrus to high-calorie cues in obese-T2DM compared with lean-T2DM, further supporting modulation of visual processing pathways.

Two studies additionally performed ‘responder’ subgroup analyses, demonstrating that neural responsivity differed according to subsequent ad libitum caloric-intake reductions following acute GLP-1RA administration. Schlögl et al. (2013) reported increased hypothalamic eigen-vector centrality during food cue exposure only among individuals who subsequently reduced caloric intake following GLP-1RA infusion, while ‘nonresponders’ demonstrated no significant effects. Bae et al. (2019) also observed differential treatment-related activation patterns across posterior cingulate, medial frontal, hypothalamic, temporal, and orbitofrontal regions, such that obese ‘responders’ reduced activation whereas lean ‘responders’ increased activation during food cue exposure.

##### 3.4.1.1. Brain-Behavior Associations.

Several studies additionally examined associations between GLP-1RA-induced CNS changes to food cues and behavioral, hormonal, or appetitive measures (Supplementary Table 1). Across food cue paradigms, these relationships varied according to metabolic status, eating behavioral phenotype, and experimental context. In individuals with obesity and obesity-T2DM, acute GLP-1RA-related changes in caloric intake positively correlated with GLP-1RA-induced changes in food cue activation in the insula, OFC, and caudate, whereas comparable associations were not observed in lean individuals (van Bloemendaal et al., 2014). Similarly, reduced eating pleasantness following 17 days of liraglutide treatment was associated with lower inferior parietal activation, whereas greater hunger and appetite correlated with increased activation across attentional and salience-related parietal and occipital regions (Farr et al., 2016b). Nausea showed an inverse association with cingulate activation (Farr et al., 2016b). Bae et al. (2019) additionally reported positive associations between satiety/fullness measures and hypothalamic activation during high-calorie food cue exposure, while inverse associations were observed between visual cortical activation and post-fMRI satiety and craving.

Baseline eating behavior phenotype also appeared to influence GLP-1RA-related CNS responsivity (van Bloemendaal et al., 2015a; van Ruiten et al., 2022a). In individuals with obesity and obese-T2DM, higher emotional eating scores were associated with less pronounced attenuation of insula and amygdala activation following acute exenatide administration, whereas these associations were absent in lean individuals (van Bloemendaal et al., 2015a). Similarly, higher emotional and external eating scores were associated with reduced attenuation of amygdala, caudate, insula, and OFC activation following sub-acute liraglutide treatment (10 days), but these associations were no longer observed at 12 weeks (van Ruiten et al., 2022a). In contrast, higher restrained eating scores were associated with greater reductions in insula and caudate activation following longer-term treatment (12 weeks) (van Ruiten et al., 2022a).

Further, associations between hormones and CNS activation were reported during food cue exposure. Farr et al. (2016a) reported that liraglutide decreased leptin levels while increasing GIP, and these changes were in turn associated with responses across the insula, thalamic, parietal, and prefrontal regions. These findings suggest that liraglutide’s effects in peripheral hormones modulate the CNS reactivity to food cues (Farr et al., 2016a).

##### 3.4.1.2. CNS effects from non-food cue paradigms.

Evidence from non-food cue paradigms is limited. In individuals with AUD, 26 weeks of exenatide treatment reduced cue-induced activation within all reward-related ROIs, including the ventral and dorsal striatum, NAc, putamen and caudate. Exploratory whole brain analysis further identified reduced activation within frontal, temporal, septal, hippocampal, and parahippocampal regions (Klausen et al., 2022b). Despite these neural effects, exenatide did not significantly reduce heavy drinking days or total alcohol intake in the overall sample. However, exploratory subgroup analysis indicated possible moderation by BMI, with individuals with comorbid AUD-obesity (BMI > 30) demonstrating reductions in heavy drinking days whereas lean (BMI < 25) individuals with AUD showed increases following treatment. In contrast, in individuals with primary polydipsia, dulaglutide treatment did not significantly alter neural responses to beverage cues in, despite concurrent reductions in fluid intake (Winzeler et al., 2021).

##### 3.4.1.3. Summary of cue reactivity findings.

Overall, cue reactivity paradigms demonstrated variable CNS effects depending on cue contrast, feeding state, treatment duration, and study population. Nevertheless, a relatively consistent finding across studies was attenuation of reward-related responsivity to highly desirable or high-calorie cues following acute or shorter-term GLP-1RA administration, particularly within insula, striatum, amygdala, and OFC. In contrast, findings following longer-term treatment were more variable, with attenuation or loss of earlier CNS effects over time. Additional involvement of hypothalamic, frontal, parietal, temporal, and visual regions further suggested that GLP-1RA-related effects may extend beyond canonical reward pathways to broader homeostatic, attentional, and sensory processing systems. Together, these findings suggest that GLP-1RAs modulate distributed neural systems involved in cue-driven motivation and appetitive behavior, although the magnitude and direction of these effects appear to be influenced by treatment duration, feeding state, metabolic status, and behavioral phenotype (Fig. 3).

#### 3.4.2. Task-based fMRI: anticipation and consumption of food

Six studies examined the effects of GLP-1RA medications on anticipatory (‘wanting’) and consummatory (‘liking’) phases of reward processing (Supplementary Table 1). Most were conducted in fasted individuals with obesity and/or T2DM, and one study investigated individuals with PCOS under both fasted and postprandial conditions (Jensterle et al., 2025). Four used ROI-based analyses focused on the insula, putamen, caudate, amygdala and OFC (van Ruiten et al., 2022a, 2022d; ten Kulve et al., 2016; van Bloemendaal et al., 2015b), whereas two utilized whole-brain approaches (Jensterle et al., 2025; Coppin et al., 2023).

Across paradigms, GLP-1RA-related effects on reward anticipation and consumption appeared variable and frequently dependent on metabolic status and treatment duration. In an acute exenatide study comparing obese-T2DM, normoglycemic obesity, and lean normoglycemic individuals, treatment-related modulation differed across groups and reward phases (van Bloemendaal et al., 2015b). Individuals with obese-T2DM demonstrated reduced activation during anticipation but increased activation during consumption within the putamen, insula, and amygdala.In lean individuals, activation was reduced in the OFC during anticipation and increased in the caudate during consumption. Normoglycemic obese individuals showed increased OFC activation during consumption but no significant anticipation-related effects. Importantly, pretreatment with the GLP-1R antagonist exendin 9–39 largely blocked these effects, supporting GLP-1 receptor specificity (van Bloemendaal et al., 2015b).

Studies investigating GLP-1RA treatment across multiple treatment timepoints demonstrated significant variability in CNS response despite consistent improvements across metabolic outcomes. van Ruiten et al. (2022d) reported no exenatide-related neural effects during either anticipation or consumption after 10 days of treatment in obese-T2DM individuals. However, after 16 weeks of treatment, reduced anticipation-related activation in the insula and increased consumption-related activation in the caudate emerged. In contrast, ten Kulve et al. (2016) observed the opposite temporal pattern, with increased caudate and insula activation following 10 days of liraglutide treatment that was no longer evident after 12 weeks.

Other studies reported minimal or condition-dependent effects. Coppin et al. (2023) found no significant differences between liraglutide and placebo following 16 weeks of treatment in obesity. Similarly, 16 weeks of semaglutide treatment did not significantly alter neurofunctional responses to glucose versus water tasting in women with PCOS under fasted conditions, despite improvements in taste recognition sensitivity and metabolic outcomes (Jensterle et al., 2025). However, under postprandial conditions, semaglutide-treated participants demonstrated increased angular gyrus activation during glucose tasting (Jensterle et al., 2025).

##### 3.4.2.1. Brain-behavior associations.

Across studies, activation within reward processing regions, including the insula, putamen, caudate, amygdala, and vmPFC, was associated with BMI, weight change, food liking, and eating behavior phenotypes (van Ruiten et al., 2022a, 2022d; ten Kulve et al., 2016; van Bloemendaal et al., 2015b). Specifically, following acute exenatide administration, BMI was positively associated with putamen activation during anticipation, but inversely correlated during consumption (van Bloemendaal et al., 2015b). Increased insula activation following 10 days of liraglutide treatment was additionally associated with greater subsequent weight change (ten Kulve et al., 2016). Independent of GLP-1RA treatment, greater subjective liking of a milkshake was also associated with increased vmPFC and amygdala activation during reward consumption (Coppin et al., 2023).

Associations between CNS responsivity and eating behavior phenotypes were additionally reported within reward-related circuitry (van Ruiten et al., 2022a). Higher emotional and external eating scores were associated with anticipatory and consummatory reward-related activation at baseline, although these relationships were no longer significant following liraglutide treatment at either 10 days or 12 weeks. In contrast, higher restrained eating was associated with greater reductions in bilateral caudate activation during reward anticipation following 12 weeks of liraglutide treatment, suggesting greater sensitivity to GLP-1RA-related modulation of reward circuitry in individuals with higher baseline restrained eating (van Ruiten et al., 2022a).

##### 3.4.2.2. Summary of anticipation and consumption findings.

Although findings were heterogeneous, particularly with respect to the duration and persistence of CNS effects, several studies demonstrated a relatively consistent pattern of reduced activation during food anticipation and increased activation during consumption with GLP-1RA (Fig. 4). This dissociation may reflect differential modulation of anticipatory (‘wanting’) versus consummatory (‘liking’) components of reward processing by GLP-1RAs, suggesting that these agonists may not globally suppress reward-related responses but may instead differentially modulate distinct components of reward. Additionally, the reviewed studies suggest that CNS responses to GLP-1RA treatment may vary according to baseline metabolic status, feeding state during scanning (fasted versus postprandial), and individual differences in baseline eating behaviors (van Ruiten et al., 2022a, 2022d; ten Kulve et al., 2016; van Bloemendaal et al., 2015b).

#### 3.4.3. Task-Based fMRI: Olfactory Paradigms

Three studies examined CNS responsivity using olfactory paradigms comparing lavender scent exposure with fresh air following GLP-1RA treatment (Supplementary Table 1). All were conducted in obese-T2DM and evaluated the effects of intermediate-term treatment with liraglutide (Cheng et al., 2022; Li et al., 2025; Zhang et al., 2019), with one additionally evaluating exenatide (Zhang et al., 2019). Treatment duration was 16 weeks in two studies (Cheng et al., 2022; Li et al., 2025), and 3 months in the third (Zhang et al., 2019). All studies used ROI analysis, focusing either on the primary olfactory cortex (Li et al., 2025) or on a broader ‘olfactory network’ comprising primary olfactory regions and additional areas implicated in reward, salience attribution, interoception, and memory, including the OFC, insula, hippocampus, and parahippocampus (Cheng et al., 2022; Zhang et al., 2019).

In the studies investigating the broader olfactory network, increased activation following intermediate-term liraglutide or exenatide treatment relative to baseline was observed in parahippocampus and hippocampus during lavender scent exposure (Cheng et al., 2022; Zhang et al., 2019). However, in Zhang et al. (2019), these effects were no longer significant following additional adjustment for BMI and HbA1c, suggesting that the observed effects may have partially been attributable to improvements in glycemic control and weight loss. Both studies additionally reported improvements in cognitive performance, including memory, attention, executive function, and olfactory identification following treatment (Cheng et al., 2022; Zhang et al., 2019). Cheng et al. (2022) further demonstrated that increased hippocampal activation partially mediated improvements in delayed memory performance. In contrast, while Li et al. (2025) similarly observed improvements in cognitive and metabolic outcomes post liraglutide treatment, no significant changes in odor-induced functional connectivity were identified relative to baseline in individuals with T2DM.

Overall, findings from fMRI olfactory paradigms provide preliminary evidence that GLP-1RA treatment may modulate olfactory-related limbic and memory circuitry along with concurrent cognitive and metabolic improvement. However, these findings should be interpreted cautiously given the limited number of studies and small sample sizes.

#### 3.4.4. Task-based fMRI: Memory and Learning Paradigms

Two studies examined the effects of GLP-1RAs on executive function-related neural activity using memory and learning paradigms (Klausen et al., 2022b; Hanssen et al., 2023) (Supplementary Table 1).

Klausen et al. (2022b) investigated working memory-related activation during an N-back task in individuals with AUD following 26 weeks of exenatide treatment. Relative to placebo, exenatide-treated participants demonstrated reduced activation within the right frontal pole and superior frontal gyrus during higher working memory load, with no additional differences in exploratory whole-brain analyses.

Hanssen et al. (2023) evaluated associative learning during acute liraglutide administration in insulin-sensitive and insulin-insensitive individuals. Liraglutide selectively increased adaptive prediction-error encoding in insulin-insensitive individuals within the ventromedial PFC and dorsal ACC, whereas insulin-sensitive individuals demonstrated no significant neural changes. Insulin-insensitive individuals additionally demonstrated liraglutide-related improvements in learning rate and adaptive prediction error processing as compared with placebo.

These preliminary findings suggest that GLP-1RA treatment may influence memory- and reinforcement-learning-related neural processes. However, the limited number of studies and heterogeneity in paradigms, populations, and treatment durations preclude definitive conclusions and warrant further investigation in larger, well-powered studies.

#### 3.4.5. Resting-state fMRI

Seven studies investigated the effects of GLP-1RAs on resting-state brain function across diverse clinical populations (Winzeler et al., 2021; Binda et al., 2019; Cheng et al., 2022; Zhang et al., 2019; Coveleskie et al., 2017; Verovnik and Vovk, 2024; Watson et al., 2019) (Supplementary Table 2). Most used seed-based functional connectivity approaches to examine circuitry implicated in appetite regulation, reward processing, sensory integration, and cognition, whereas one additionally evaluated resting-state BOLD signal intensity across the cortical surface (Binda et al., 2019).

Two studies examining acute GLP-1RA administration demonstrated effects involving hypothalamic–brainstem and sensory-related networks. In individuals with obesity, acute exenatide increased functional connectivity between the NTS, hypothalamus, and thalamus, whereas lean individuals demonstrated no significant effects relative to placebo (Coveleskie et al., 2017). Binda et al. (2019) reported widespread increases in resting-state BOLD signal intensity across visual and cortical regions following acute exenatide administration independent of obesity status. In contrast, obesity was associated with reduced resting-state signal within visual cortical regions and the insula, independent of treatment.

Longer-term GLP-1RA administration was associated with more variable findings. In individuals with subjective cognitive complaints, liraglutide treatment increased hippocampal connectivity with frontal, posterior cingulate/precuneus, and occipital/parietal regions following 12 weeks of treatment relative to placebo, despite no corresponding improvement in cognitive outcomes (Watson et al., 2019). Semaglutide treatment in women with PCOS was associated with within-group increases in connectivity between the amygdala and insula and between the amygdala and parahippocampus following 16 weeks of treatment, alongside reduced connectivity between bilateral medial temporal regions and between the precuneus and insula (Verovnik and Vovk, 2024). However, no significant between-group effects relative to placebo were identified despite significant improvements in weight and waist circumference.

In contrast, several studies reported predominantly null resting-state findings despite concurrent behavioral, metabolic, cognitive, or olfactory improvements. Dulaglutide treatment in primary polydipsia did not significantly alter resting-state connectivity despite improvements in thirst, fluid intake, and quality of life (Winzeler et al., 2021). Similarly, no significant changes in olfactory-network connectivity or resting-state metrics, including Amplitude of Low Frequency Fluctuations (ALFF) and Regional Homogeneity (ReHo), were observed following liraglutide treatment in T2DM (Cheng et al., 2022) or following liraglutide or exenatide treatment in obesity-T2DM (Zhang et al., 2019).

##### 3.4.5.1. Brain-behavior associations.

Various studies additionally reported associations between resting-state activation and behavioral or metabolic outcomes (Supplementary Table 2). Following acute exenatide administration, increases in NTS–hypothalamic and NTS-thalamic connectivity were positively associated with ad libitum caloric intake across both lean and obese individuals, whereas associations with hunger ratings differed according to obesity status (Coveleskie et al., 2017). At baseline, greater NTS-thalamic connectivity was positively associated with hunger ratings in lean relative to obese individuals. However, following acute exenatide, the positive association between increased NTS-hypothalamic connectivity and hunger was stronger in obese relative to lean individuals (Coveleskie et al., 2017). Binda et al. (2019) additionally demonstrated that higher BMI was associated with lower resting-state BOLD signal following acute exenatide administration, suggesting that adiposity may influence resting-state CNS responsivity to GLP-1RA treatment. Notably, several cognitive and olfactory improvements reported in longer-term studies were no longer significant after adjustment for HbA1c or BMI, suggesting that concurrent glycemic and anthropometric changes may have contributed to these outcomes (Cheng et al., 2022; Zhang et al., 2019).

##### 3.4.5.2. Summary of resting-state findings.

Overall, the resting-state fMRI literature suggests that GLP-1RA treatment may influence connectivity within homeostatic, limbic, hippocampal, and reward-related networks, although findings varied considerably in direction, magnitude, and persistence across studies. Reported effects primarily consisted of within-group connectivity changes, drug or group main effects, or associations between resting-state measures and behavioral or metabolic outcomes, whereas only one investigation reported significant between-group effects relative to placebo (Watson et al., 2019). Notably, nearly half of the reviewed resting-state studies reported null findings despite concurrent cognitive, behavioral, or metabolic improvement (Winzeler et al., 2021; Cheng et al., 2022; Zhang et al., 2019). Together, the limited and heterogeneous resting-state literature provides preliminary evidence that GLP-1RAs may modulate intrinsic functional connectivity, particularly within networks implicated in appetite regulation and reward processing. However, larger longitudinal and randomized studies are needed to clarify the reproducibility, generalizability, and clinical relevance of these effects.

### 3.5. Structural neuroimaging

Four studies investigated the neuroanatomical effects of GLP-1RAs using structural MRI across neurodegenerative, psychiatric and metabolic populations (Li et al., 2025; Edison et al., 2026; Mansur et al., 2017; Mullins et al., 2019) (Supplementary Table 3).

Two studies examined the neuroprotective effects of longer-term GLP-1RA treatment in neurodegenerative populations (Edison et al., 2026; Mullins et al., 2019). Mullins et al. (2019) evaluated 18 months of exenatide treatment in individuals with mild cognitive impairment or mild Alzheimer’s disease and observed progressive gray matter atrophy over time in both groups, with no significant differences in gray matter volume or cortical thickness relative to placebo. Although exenatide-treated participants demonstrated reductions in BMI and glucose levels, cognitive decline progressed similarly across groups over the study period. In contrast, Edison et al. (2026) reported evidence suggestive of attenuated structural decline following 52 weeks of liraglutide treatment in individuals with mild-to-moderate Alzheimer’s disease. Relative to placebo, liraglutide-treated participants demonstrated smaller reductions in total gray matter volume and temporal, frontoparietal, and parietal volumes. Voxel-based morphometry analyses further indicated slower decline across whole gray matter and white matter and within hippocampal, frontal, medial temporal, parietal and temporal cortices. Liraglutide-treated individuals additionally demonstrated slower decline on cognitive and executive functioning subscales, suggesting the potential for liraglutide to attenuate both longitudinal structural atrophy and cognitive decline in Alzheimer’s disease (Edison et al., 2026).

Beyond studies in patients with neurodegenerative diseases, Mansur et al. (2017) investigated liraglutide-related structural alterations in those with diagnosed mood disorders and co-occurring executive dysfunction. Following 4 weeks of treatment, increased gray matter volume was observed in reward- and executive-function-related regions, including the NAc, putamen, pallidum, rostral middle frontal cortex, and lateral OFC. Associations between GLP-1RA treatment-related changes in gray matter volume, BMI, and cognitive performance were additionally observed, although the direction of these relationships varied across regions. Li et al. (2025) observed no significant structural changes within the primary olfactory cortex following 16 weeks of liraglutide treatment in individuals with T2DM, despite concurrent improvements in glycemic control, anthropometric measures, olfaction, executive functioning, memory, and attention.

Overall, the structural MRI literature remains limited and heterogeneous with respect to clinical populations, neuroanatomical targets, and analytic approaches. Although the available evidence suggest the potential for GLP-1RAs to attenuate structural decline or modulate gray matter volume in select populations, findings remain inconsistent and should be interpreted cautiously, particularly given that several significant effects were identified using uncorrected statistical analyses. Larger, well-powered longitudinal studies are necessary to clarify whether GLP-1RAs have neuroprotective and neurorestorative effects on brain structure.

### 3.6. Molecular Imaging

Seven studies used either PET or SPECT to investigate GLP-1RA-related effects on cerebral glucose metabolism (n = 4), amyloid burden (n = 1), cerebral perfusion (n = 1), and dopaminergic signaling (n = 3), with some evaluating more than one outcome (Supplementary Table 4). The reviewed studies were conducted across a diverse range of clinical populations including Alzheimer’s disease (AD), Parkinson’s disease (PD), AUD, individuals with mild postprandial hyperglycemia, and healthy controls. Analyses focused on a range of a priori-defined cortical and hippocampal regions implicated in neurodegeneration and executive function, and homeostatic and reward-related circuitry, including the hypothalamus, limbic regions, and striatum.

#### 3.6.1. CNS effects of GLP-1RA on brain glucose metabolism

Four studies investigated the modulatory effect of GLP-1RAs on brain glucose metabolism as measured with [18 F]-fluorodeoxyglucose-PET ([^18^F]-FDG) (Edison et al., 2026; Daniele et al., 2015; Gejl et al., 2016, 2017), three of which were conducted in individuals with AD. Different metrics were used to quantify brain glucose metabolism across studies, including cerebral glucose metabolic rate (CMRglc), standard uptake value (SUV), and kinetic measures of brain glucose uptake and clearance.

In a 26-week placebo-controlled study of mild AD, Gejl et al. (2016) demonstrated preservation of CMRglc following liraglutide treatment, whereas placebo-treated individuals showed progressive reductions in metabolism across the cerebellum, precuneus, parietal, temporal, and occipital regions. Exploratory follow-up analyses in the same cohort demonstrated liraglutide-related increases in brain glucose uptake and clearance, as well as normalization of cortical Tmax values, reflecting altered blood-brain glucose transfer capacity over time (Gejl et al., 2017). At baseline, several FDG-derived metabolic markers were additionally associated with AD duration and cognitive performance, with longer disease duration correlated with lower cerebral metabolic activity and glucose uptake (Gejl et al., 2017). After 26 weeks of liraglutide treatment, individuals with AD demonstrated significant decreases in weight, blood pressure, and fasting plasma glucose compared with placebo, however, no significant changes were observed regarding cognitive functioning (Gejl et al., 2016;Gejl et al., 2017).

In contrast, Edison et al. (2026) reported no significant differences in FDG SUV measures following 52 weeks of liraglutide treatment in mild-to-moderate AD. Despite the absence of significant brain metabolic differences, liraglutide-treated participants demonstrated slower decline in cognitive and executive functioning measures relative to placebo.

Beyond studies in AD populations, Daniele et al. (2015) examined the acute effects of exenatide on CMRglc during oral glucose administration in individuals with mild postprandial hyperglycemia. Acute administration increased CMRglc across total gray matter and the cortex, as well as within predefined glucose-homeostasis and food-reward ROIs. In contrast, hypothalamic CMRglc was reduced. Metabolic activity across several cortical and limbic regions was additionally correlated with peripheral glucose flux measures (rate of glucose absorption/appearance) following exenatide administration, suggesting coupling between central metabolic responses and systemic glucose regulation.

In addition to investigating brain glucose metabolism, Gejl et al. (2016) used [11]C-labeled Pittsburgh Compound-B PET ([^11^C]-PIB PET]) to assess beta-amyloid plaque accumulation, a hallmark of AD, using PIB binding potential (PIB-BPnd). The study also evaluated a surrogate of cerebral blood flow (sCBF) within the same AD cohort treated with liraglutide or placebo for 26 weeks. PIB-BPnd increased over time in temporal and occipital regions in both liraglutide-treated participants and placebo-treated individuals, suggesting no clear treatment-related effect on amyloid accumulation. Regarding sCBF, no significant liraglutide-related changes were observed over the course of treatment. In contrast, the placebo group demonstrated increased sCBF within frontal, parietal, occipital, and cerebellar regions, as well as globally across the cortex. These findings may suggest that liraglutide treatment stabilized cerebral perfusion-related changes relative to the progressive alterations observed in the placebo group. However, interpretation should remain cautious given the exploratory nature of the analysis and the lack of correction for multiple comparisons.

#### 3.6.2. CNS effects of GLP-1RA on Dopaminergic Function

Three SPECT imaging studies investigated the effect of GLP-1RA on dopamine transporter (DAT) availability, which serve as biomarkers of dopaminergic terminals (Klausen et al., 2022b; Athauda et al., 2017; Jensen et al., 2020).

In healthy controls, acute exenatide administration did not significantly alter DAT binding potential, suggesting no immediate GLP-1RA-related effects on dopamine transporter availability in this population (Jensen et al., 2020). In contrast, longer-term treatment with exenatide was associated with alterations in DAT availability in two different clinical populations. In a SPECT substudy within a larger randomized clinical trial of AUD, Klausen et al. (2022b) observed no baseline differences in DAT binding potential between AUD and healthy controls. However, following 26 weeks of exenatide, striatal DAT binding potential was reduced relative to baseline. The authors suggested that this may reflect GLP-1RA-induced compensatory modulation of dopaminergic dysfunction in AUD (Klausen et al., 2022b). In this study, craving appeared to normalize relative to controls over time, with significantly greater craving in the AUD group at baseline but no significant between-group differences observed after treatment (Klausen et al., 2022b). Craving changes were not associated with DAT measures, though the small sample size that underwent SPECT imaging limited power to identify effects.

In PD, Athauda et al. (2017) demonstrated slower decline in striatal DAT availability following long-term exenatide treatment over 60 weeks (48 weeks on treatment and 12 weeks off) relative to placebo. These imaging findings paralleled improvements in motor symptom severity over time. However, no significant effects were observed across broader cognitive or non-motor outcomes.

#### 3.6.3. Molecular imaging of GLP-1 receptors in the human brain

We identified one PET study that used ^68^Ga-NODAGA-exendin-4 with PET in obese subjects with the aim to measure brain GLP-1Rs (Deden et al., 2021). The study found significant binding in the pituitary area, which is a region with a high concentration of GLP-1Rs but it’s also a region devoid of blood brain barrier. In contrast no binding was observed in any other brain areas consistent with minimal radiotracer passage through the blood brain barrier (Deden et al., 2021). As such the authors concluded that ^68^Ga-NODAGA-exendin-4 was an inadequate radiotracer for quantifying GLP-1 receptors in brain.

#### 3.6.4. Summary of molecular imaging findings

Overall, the reviewed PET and SPECT studies suggest that GLP-1RA treatment may modulate brain glucose metabolism and striatal dopamine transporter availability across various clinical populations. However, findings varied substantially across investigations with respect to tracer selection, quantification measures, and study populations, with two of seven studies reporting predominantly null findings. Consequently, the current molecular imaging literature remains insufficient to definitively characterize the consistency or specificity of GLP-1RA-related effects on brain metabolism or DAT availability.

AD was the most frequently investigated disorder within the PET/SPECT literature, although findings across the AD cohorts were mixed. In one AD cohort, liraglutide treatment attenuated the expected decline in brain glucose metabolism and improved measures of blood-brain glucose transport capacity over time (Gejl et al., 2016, 2017), suggesting potential neuroprotective metabolic effects. In contrast, a larger cohort of individuals with mild-to-moderate AD demonstrated no significant treatment-related differences in FDG-PET metabolic measures following 52 weeks of liraglutide despite evidence for slower cognitive decline (Edison et al., 2026).

Beyond AD, a PET study suggested increased glucose metabolism within homeostatic and reward-related regions following acute GLP-1RA administration in mild postprandial hyperglycemia (Daniele et al., 2015). SPECT studies showed reduced striatal DAT availability following longer-term treatment in AUD and reduced DAT decline in PD (Klausen et al., 2022b; Athauda et al., 2017).

Together, these findings highlight the potential utility of molecular imaging approaches for characterizing GLP-1RA-related metabolic and neurochemical CNS effects. However, the current PET/SPECT literature remains sparse and methodologically heterogeneous, underscoring the need for larger and more standardized investigations across samples. There is also a need for brain-penetrant radiotracers that can be used to measure GLP-1 receptors in the human brain. Such tracers would enable assessment of GLP-1R occupancy by different GLP-1RA medications and determine whether GLP-1R expression varies across clinical populations and disease states.

### 3.7. Intracranial EEG

Choi et al. (2025) leveraged intracranial electroencephalography (iEEG) recordings from an ongoing early feasibility trial of responsive deep brain stimulation (rDBS) for treatment-refractory obesity and loss-of-control eating (Supplementary Table 5). The study examined a unique case of tirzepatide-related modulation of electrophysiological activity within the human NAc. The analysis included three individuals with persistent obesity and loss-of-control eating following bariatric surgery: one receiving tirzepatide treatment and two control participants used as reference cases (Choi et al., 2025).

Across the two rDBS control participants, increased NAc delta–theta band power (≤7 Hz) was identified during periods of severe food preoccupation relative to control states (periods without severe food preoccupation), suggesting a potential biomarker of food preoccupation. In contrast, in the tirzepatide-treated patient, this NAc delta–theta biomarker was no longer evident during post-implantation months 2-4, a period of tirzepatide dose escalation, as delta-theta band power during severe food preoccupation was indistinguishable from that observed during control states. This change coincided with a prolonged reduction in severe food preoccupation episodes during the same period. However, during months 5–7, despite treatment at the maximum tirzepatide dose, the NAc delta–theta biomarker re-emerged alongside the recurrence of severe food preoccupation episodes, which subsequently increased in frequency over time. These findings provide preliminary electrophysiological evidence that GLP-1RA treatment may modulate food reward-related neural activity within the human NAc, while also suggesting that early CNS effects may attenuate during prolonged treatment (Choi et al., 2025).

## 4. Discussion

This systematic review synthesized available clinical neuroimaging evidence on the CNS effects of GLP-1RAs across multiple imaging modalities and experimental paradigms. Collectively, the reviewed literature suggests that GLP-1RAs are associated with modulation of distributed CNS systems involved in reward processing, appetitive regulation, homeostatic control, salience attribution, and interoceptive processing. Several potentially convergent patterns emerged, including modulation of homeostatic and appetite-regulating circuitry, attenuation of reward-related responses following acute GLP-1RA administration, and preliminary indications that CNS effects may evolve over the course of treatment. These effects also appeared to vary according to metabolic status and eating behavioral phenotype. Although preliminary, these findings provide insight into the neurobiological mechanisms through which GLP-1RAs may influence appetitive regulation, as well as the factors that may contribute to variability in treatment response.

### 4.1. Modulators and predictors of CNS responsivity

Several studies suggested that GLP-1RA-related neural effects vary according to behavioral, metabolic or anthropometric characteristics. In addition, subgroup analyses identified distinct patterns of CNS responsivity among individuals demonstrating differential treatment response following GLP-1RA administration. Although these findings are preliminary and derived from relatively small cohorts, they collectively suggest that interindividual variability may represent an important feature of GLP-1RA-related CNS modulation and its downstream behavioral and clinical effects.

#### 4.1.1. Eating behavior phenotypes

Preliminary evidence suggests that baseline eating behavior phenotypes may modulate the CNS effects to GLP-1RA treatment, particularly within reward-related circuitry. Specifically, individuals with higher emotional or external eating generally demonstrated less pronounced GLP-1RA-related attenuation of food reward processing, whereas higher restrained eating was associated with greater CNS attenuation following treatment (van Bloemendaal et al., 2015a; van Ruiten et al., 2022a). These findings suggest that underlying emotionally or externally driven eating tendencies may be associated with reduced CNS sensitivity to GLP-1RA treatment, whereas restrained eating may enhance sensitivity.

Notably, these neuroimaging observations broadly parallel findings from the wider clinical literature. Individuals characterized by pre-dominant restrained eating patterns demonstrated greater GLP-1RA-related weight loss, whereas emotional and external eating tendencies were associated with reduced treatment efficacy (de Boer et al., 2016). Similar associations between emotional eating and reduced weight loss were also observed following longer-term GLP-1RA therapy (Koide et al., 2025), although findings regarding external and restrained eating were less consistent. Although preliminary, the alignment between the reviewed neuroimaging findings and broader clinical literature suggests that eating behavior phenotype may meaningfully influence CNS responsivity to GLP-1RA treatment and contribute to interindividual variability in treatment outcomes.

#### 4.1.2. Metabolic Status and Adiposity as Potential Modulators of CNS Responsivity

Clinical and observational studies have reported greater weight loss following GLP-1RA treatment among individuals with higher baseline BMI or obesity-related metabolic dysfunction, whereas lower BMI has been associated with reduced treatment efficacy (Apostolopoulou and Koufakis, 2025; Crabtree et al., 2023). Consistent with this, emerging neuroimaging findings may provide preliminary mechanistic insight into how adiposity and metabolic dysfunction influence GLP-1RA-related CNS responsivity.

Preliminary findings suggest that GLP-1RA-related CNS responses differ according to metabolic and anthropometric status. Across food-cue paradigms, individuals with obesity and/or T2DM generally demonstrated greater GLP-1RA-induced modulation of food reward- and homeostatic-related circuitry following acute administration compared with lean comparators (van Bloemendaal et al., 2014; Eldor et al., 2016; Bae et al., 2019). Similar patterns were observed during reinforcement learning, where GLP-1RA-related modulation of adaptive prediction-error signaling was more pronounced in insulin-insensitive obese individuals than lean, insulin-sensitive individuals (Hanssen et al., 2023). Findings also suggest that adiposity may differentially influence the anticipatory and consummatory phases of food experience and their related wanting and liking components (van Bloemendaal et al., 2015b). Resting-state findings further suggest that GLP-1RA-related changes in NTS connectivity with homeostatic and appetite-regulating circuitry may be greater in obesity relative to lean individuals (Coveleskie et al., 2017).

Together, these findings suggest that baseline adiposity and metabolic dysfunction may influence how reward-, appetitive-, and homeostatic-related brain systems respond to GLP-1RAs. However, these effects were not consistently observed across studies, and much of the current evidence derives from relatively small acute pharmacologic paradigms. Larger, well-powered longitudinal investigations are needed to clarify the reproducibility and clinical relevance of these patterns, as well as how they evolve with sustained treatment. Such work should also determine whether similar CNS responsivity patterns extend to disorders not primarily characterized by metabolic dysfunction.

#### 4.1.3. Neural Correlates of GLP-1RA ‘Responders’

Two subgroup analyses classified participants as “responders” based on reductions in ad libitum caloric intake following acute GLP-1RA exposure. These analyses identified distinct patterns of hypothalamic and reward activation during food cue processing, either relative to ‘nonresponders’ or according to obesity status (Schlögl et al., 2013; Bae et al., 2019). Among ‘responders’, greater hunger and craving were additionally associated with stronger posterior cingulate and medial frontal activation to high-calorie food cues, potentially reflecting increased sensitivity to the satiety-enhancing effects of acute GLP-1RA administration (Bae et al., 2019).

Importantly, responders with obese-T2DM and lean-T2DM demonstrated comparable reductions in caloric intake following acute GLP-1RA administration, despite exhibiting distinct patterns of CNS activation (Bae et al., 2019). These findings suggest that metabolic and anthropometric status may influence the neurofunctional mechanisms underlying GLP-1RA responsivity, even when overt behavioral outcomes appear similar. Although preliminary, these responder analyses highlight the potential utility of neuroimaging for identifying neural markers of GLP-1RA responsivity that may not be detectable through behavioral measures alone. Future longitudinal studies will be important for determining whether these early CNS patterns are replicable and relate to longer-term treatment response and clinical outcomes.

### 4.2. Temporal dynamics of CNS effects

Across the reviewed literature, GLP-1RA-related CNS effects appeared highly dependent on treatment duration. Across several imaging modalities and paradigms, acute GLP-1RA administration and short-term treatment were most consistently associated with attenuated activation of reward- and homeostatic-related brain systems (Farr et al., 2016b; van Ruiten et al., 2022b, 2022c; Martin et al., 2025; Binda et al., 2019; ten Kulve et al., 2016; van Bloemendaal et al., 2015b; Coveleskie et al., 2017; Daniele et al., 2015). Additionally, attenuated CNS activation following acute or short-term GLP-1RA exposure was associated with appetitive and behavioral outcomes, supporting the clinical relevance of these early neural effects (Farr et al., 2016b; Bae et al., 2019; ten Kulve et al., 2016; Coveleskie et al., 2017).

Several longitudinal neuroimaging studies suggested that early GLP-1RA-related CNS effects attenuated or were no longer detectable following prolonged treatment, despite continued weight loss and metabolic improvements (ten Kulve et al., 2015; van Ruiten et al., 2022b; Martin et al., 2025; Cheng et al., 2022; Li et al., 2025; Zhang et al., 2019; Verovnik and Vovk, 2024; Edison et al., 2026; Mullins et al., 2019). While these findings may indicate that early CNS modulation contributes more strongly to weight-loss initiation than maintenance (ten Kulve et al., 2015, 2016), several studies also suggested adaptive neural responsivity during sustained GLP-1RA exposure. Reported patterns included “counter-regulatory” re-engagement of reward-related circuitry and re-emergence of neural “food preoccupation” signatures over the course of treatment (Farr et al., 2019; Choi et al., 2025). Together, these observations raise the possibility that neural adaptations during prolonged GLP-1RA treatment may contribute to reduced appetitive benefits and/or the emergence of weight-loss plateaus over time, which are common during GLP-1RA therapy and weight-loss interventions more broadly (Hall, 2024; Jastreboff et al., 2022; Wilding et al., 2021). Although preliminary, these dynamic CNS patterns may help contextualize clinical observations of a possible tolerance-like effect during prolonged GLP-1RA treatment, whereby satiety-related benefits diminish and cravings or food preoccupation re-emerge over time (Tronieri et al., 2020; Wharton et al., 2023).

These temporal patterns highlight the broader utility of neuroimaging for understanding how CNS responses to GLP-1RAs evolve over the course of treatment. Interpretation, however, remains constrained by small sample sizes, methodological heterogeneity, and limited long-term imaging data. As such, it remains unclear whether the observed patterns are stable and reproducible, reflect neural adaptation or counter-regulatory processes, or instead represent indirect effects related to ongoing metabolic and behavioral changes. Importantly, most available evidence has been derived from individuals with obesity and/or metabolic dysregulation, limiting understanding of how these evolving CNS effects may generalize to other clinically relevant populations. Emerging evidence further suggests that the temporal evolution of GLP-1RA-related CNS effects may itself vary by behavioral phenotype (van Ruiten et al., 2022a), highlighting potentially meaningful interindividual differences in treatment-related temporal dynamics. Better characterization of these neural trajectories may therefore help clarify how CNS responsivity emerges and differs between early and prolonged phases of GLP-1RA treatment and across phenotypes. Such findings may additionally provide insight into mechanisms underlying attenuation of appetitive benefits and the development of weight-loss plateaus over time.

### 4.3. Weight dependent or independent CNS effects

An important methodological and mechanistic consideration is the extent to which observed CNS effects reflect direct central actions of GLP-1R agonist-based pharmacotherapies versus indirect effects mediated by weight loss, metabolic improvement, or other peripheral mechanisms. Notably, several studies demonstrated CNS modulation following acute GLP-1RA administration (Schlögl et al., 2013; van Bloemendaal et al., 2014, 2015a; Eldor et al., 2016; Bae et al., 2019; Binda et al., 2019; van Bloemendaal et al., 2015b; Hanssen et al., 2023; Coveleskie et al., 2017; Daniele et al., 2015), indicating that GLP-1RA-related neural effects can emerge prior to substantial anthropometric change. However, because several acute administration studies also reported concurrent, albeit variable, effects on hunger or caloric intake, the extent to which these early CNS effects are independent of rapidly emerging peripheral or metabolic changes remains un-clear. Some investigations were specifically designed to minimize this confounding. For example, Farr et al. (2016a), (2016b) used a short-term treatment duration (17 days) intended to minimize confounding effects of weight loss, yet reported neural and hormonal modulation despite the absence of significant anthropometric change. Similarly, van Bloemendaal et al. (2014), (2015a) used a somatostatin pancreatic-pituitary clamp to suppress endogenous hormonal responses and control metabolic conditions, thereby better isolating GLP-1RA-induced CNS effects from secondary hormonal or metabolic changes.

Regarding longitudinal investigations, most reported CNS effects alongside concurrent improvements in body weight, glycemic status, or other metabolic indices. Despite this, associations between CNS and metabolic or anthropometric changes were not always consistent, suggesting that CNS and anthropometric responses may not always occur in parallel (Watson et al., 2019). Mechanistic interpretation is further complicated by the fact that relatively few studies statistically accounted for metabolic or anthropometric changes directly. Among those that did, several reported attenuation, loss, or emergence of CNS effects following adjustment for BMI, glycemic indices, or weight loss (Farr et al., 2019; Zhang et al., 2019; Mansur et al., 2017). This suggests that reported CNS effects may be influenced by concurrent metabolic and weight-related changes, underscoring the importance of systematically accounting for these factors in future research.

Importantly, the imaging outcomes reviewed here cannot, by themselves, establish whether the observed CNS changes reflect direct central GLP-1R engagement or indirect effects mediated via peripheral mechanisms. Determining the relevant contributions of central and peripheral pathways will therefore be important for understanding how they interact to shape neural and behavioral responses to treatment. This issue is particularly relevant as GLP-1RAs are increasingly being investigated across neurodegenerative, psychiatric, and addictive disorders, where obesity or metabolic dysfunction may not be universally present and treatment-related weight loss could, in some contexts, be harmful or potentially exacerbate other symptoms (Bartel et al., 2024).

### 4.4. Future directions and open questions

Current neuroimaging findings provide preliminary insight into how GLP-1RAs may modulate CNS systems involved in reward processing, appetitive regulation, and homeostatic control, while also raising important mechanistic and translational questions for future investigation.

Preliminary evidence suggests that GLP-1RA-related CNS effects can emerge rapidly following acute administration, differ according to feeding state (e.g., fasted versus postprandial conditions), and temporally evolve throughout treatment. These observations raise important questions regarding how treatment timing and duration of exposure influence neural responsivity to appetitive cues. For GLP-1RAs that require daily dosing, how does the timing of administration relative to meals or fasting influence reward responsivity, appetitive control, or cue-induced craving? More broadly, is the efficacy of GLP-1RA treatment influenced by circadian rhythms? Understanding these dynamics may help identify optimal administration strategies for enhancing CNS-mediated therapeutic effects and inform context-dependent therapeutic approaches to attenuate cue-induced craving and appetitive responsivity in behaviorally salient or high-risk settings.

The observation that GLP-1RA-related CNS effects may emerge even under conditions designed to minimize weight loss raises important questions about how different dosing strategies and compound-specific differences in CNS penetration influence neural modulation in the absence of substantial anthropometric change. Could lower or alternative dosing strategies preserve meaningful CNS effects while minimizing weight-related consequences in populations where substantial weight loss may be undesirable? This consideration may be particularly important when extending GLP-1R agonist medications to neurological and neuropsychiatric indications in lean individuals, for whom substantial treatment-related weight loss may offer no additional therapeutic benefit and could raise additional clinical concerns (Banks, 2026; Rossi et al., 2026). Given known pharmacokinetic and pharmacodynamic differences across GLP-1R agonist therapies, understanding dose-dependent and compound-specific CNS effects represents an important future direction for the field.

As emerging dual-agonists such as tirzepatide, which engage both GLP-1 and GIP receptors, are evaluated alongside GLP-1R-selective mono-agonists such as semaglutide, clarifying compound-specific CNS effects will become increasingly important. Both GLP-1 and GIP receptor systems are expressed within the CNS, including regions implicated in homeostatic, reward, and cognitive processes (Nyberg et al., 2005; Zhang et al., 2021). Preclinical evidence further indicates that central GIP receptor signaling can independently influence food intake and body-weight regulation (Zhang et al., 2021). Accordingly, CNS effects observed with tirzepatide may reflect contributions from GLP-1R and GIP receptor signaling, as well as interactions between these receptor systems (Zhang et al., 2021; Drucker, 2026). Such effects should therefore not be attributed solely to GLP-1R activation. Tirzepatide has also demonstrated greater improvements in glycemic control and weight loss than currently available GLP-1R mono-agonists (Aronne et al., 2025; Frías et al., 2021), further complicating attribution of differential CNS effects to a specific receptor system. Notably, one of the two studies examining tirzepatide in this review included direct comparisons with both liraglutide (a GLP-1R mono-agonist) and placebo (Martin et al., 2025). At six weeks, neither treatment significantly differed from placebo in neural responses to high-fat/high-sugar food cues. However, direct comparison between the active treatments showed reduced cue-related neural responses following tirzepatide relative to liraglutide. While this finding suggests a preliminary signal of differential CNS responsivity between dual- and mono-agonist treatments, it should be interpreted cautiously given the absence of significant differences from placebo and the use of uncorrected statistical thresholds. Moreover, this comparison cannot establish whether any between-treatment differences reflect GIP receptor signaling or other pharmacological differences between compounds. With only two neuroimaging studies of tirzepatide identified, further adequately powered head-to-head studies are needed to determine whether dual GIP/GLP-1R agonists and GLP-1R mono-agonists produce distinct patterns of CNS modulation. If differences are observed, their underlying mechanisms and clinical significance will require further investigation.

The reviewed neuroimaging studies were conducted predominantly in individuals with obesity or broader metabolic dysregulation. Accordingly, it remains unclear whether the observed CNS effects generalize to populations with differing metabolic, anthropometric, and clinical characteristics, or instead reflect obesity-related pathophysiological mechanisms. In individuals with obesity, GLP-1R agonist medications may act upon neural systems already altered by obesity and metabolic dysfunction, potentially leading to CNS responses and treatment effects that differ from those observed in lean individuals. Indeed, emerging evidence synthesized in this review suggests that baseline BMI and metabolic status may influence both CNS and clinical responses to GLP-1RA treatment, although findings were not always consistent (van Bloemendaal et al., 2014, 2015b; Eldor et al., 2016; Bae et al., 2019; Klausen et al., 2022b; Hanssen et al., 2023; Coveleskie et al., 2017). These differences may be further modified by the presence of neurodegenerative or psychiatric conditions, such that CNS responses vary not only between lean and obese individuals, but also based on the interaction between metabolic status and underlying clinical diagnosis (Boswell et al., 2021). Obesity, addiction, and several psychiatric disorders share overlapping neurofunctional mechanisms related to reward processing, appetitive control, and affect regulation; however, important disorder-specific and interindividual differences within these systems have also been reported (Boswell et al., 2021; Milaneschi et al., 2019; Schulte et al., 2016; Tomasi and Volkow, 2013; Volkow et al., 2013). The extent to which these shared mechanisms are associated with comparable CNS responses to GLP-1RA treatment across populations remains unknown. Improved understanding of how these mechanistic similarities and differences relate to CNS modulation associated with GLP-1RA medications may help explain variability in treatment response and clinical outcomes, and clarify their transdiagnostic therapeutic potential.

Findings from clinical trials, observational studies, and the reviewed neuroimaging literature suggest that behavioral and neurobiological differences may influence both CNS responses to GLP-1RAs and overall treatment outcomes. Across psychiatry, neurodegenerative disorders, and obesity, growing recognition of substantial heterogeneity in underlying mechanisms has motivated efforts to identify more homogeneous clinical subgroups to improve treatment prediction and efficacy (Brucar et al., 2025a; Tozzi et al., 2024; Drossel et al., 2023; Pelin et al., 2021; Acosta et al., 2021; Mann et al., 2018; Drysdale et al., 2017; Brucar et al., 2025b, 2026). Preliminary evidence suggests that similar approaches may also be relevant for GLP-1RA treatment. For instance, distinct craving-related behavioral profiles have been associated with differences in longer-term semaglutide outcomes (Wharton et al., 2023). Accordingly, phenotypic and neuroimaging-based “subtyping” approaches aimed at identifying more mechanistically homogeneous patient groups (Brucar et al., 2023; Feczko et al., 2019; Zhang et al., 2023) may help clarify the mechanisms underlying interindividual variability in both CNS and clinical response to GLP-1RAs, while also supporting the development of more individualized treatment strategies.

The use of brain imaging tools to investigate the CNS effects of GLP-1RA is still in its infancy but offers important opportunities to help characterize treatment heterogeneity and ultimately support more personalized interventions. This will require larger studies sufficiently powered to assess whether brain responses to GLP-1RAs differ by sex, to investigate how age influences these brain responses and their associated metabolic effects, and to better understand the interaction between peripheral and central effects. Future studies should also investigate potential differences between individual GLP-1RAs and emerging combination therapies as newer compounds with greater weight-loss efficacy than current treatments become available. Broader neuroimaging approaches are also needed to establish the scope of GLP-1RA-related CNS effects. To date, task-based fMRI studies have predominantly focused on reward, food-cue, and appetitive processing, with relatively few examining other functional domains, such as cognitive, emotional, sensory or motor processes. Expanding the range of experimental paradigms will therefore be important for determining whether, and to what extent, treatment-related effects involve neural processes beyond those emphasized in the current literature. Important gaps also remain across imaging modalities. Diffusion-weighted imaging (DWI) following GLP-1RA administration remains largely unexplored and could provide complementary insight into treatment-related changes in brain microstructure, including within white matter tracts, particularly given evidence of alterations across obesity and neuropsychiatric conditions (Daoust et al., 2021; Kochunov et al., 2020). Similarly, current molecular imaging evidence remains insufficient to establish GLP-1R occupancy or direct receptor engagement in the human brain. Advances in molecular imaging, including the development and validation of brain-penetrable GLP-1R-specific radiotracers, will therefore be needed to directly characterize central GLP-1 receptor systems. Such tools could enable a more comprehensive understanding of neural mechanisms, sources of heterogeneity, and the therapeutic potential of GLP-1R agonist therapies across diverse populations. An overview of the emerging neurobiological insights, mechanistic considerations, and future research priorities identified throughout this systematic review are summarized in Fig. 5.

### 4.5. Limitations

Several limitations of the current review and broader neuroimaging literature should be acknowledged. Considerable heterogeneity across study designs, imaging modalities, task paradigms, treatment duration, feeding state during acquisition, analytic strategies, and reported outcome measures limited direct cross-study comparison and precluded quantitative synthesis. The inclusion of studies with diverse empirical designs also warrants consideration, as these designs vary in evidentiary strength and in how confidently observed neuroimaging effects can be attributed to treatment, particularly in observational and uncontrolled studies. Moreover, the available imaging evidence cannot, by itself, establish direct central GLP-1R engagement and therefore cannot determine whether observed CNS changes arise from direct central actions or indirect peripheral mechanisms. In addition, many included studies were exploratory, with relatively small sample sizes, increasing susceptibility to underpowered subgroup analyses and limiting evaluation of important sources of interindividual variability. The reviewed literature was also conducted predominantly in individuals with obesity or broader metabolic dysregulation, potentially limiting generalizability to other clinical populations. In addition, most neuroimaging studies focused on reward-related paradigms (e.g., food cue reactivity, reward anticipation and consumption), constraining our understanding of the broader effects of GLP-1RAs on neural systems and cognitive or affective processes. Accordingly, the reviewed findings should be interpreted cautiously pending larger, more standardized, and longitudinal neuroimaging investigations.

## 5. CONCLUSIONS

The current systematic review synthesized available neuroimaging evidence examining GLP-1RA-related CNS effects across multiple imaging modalities and experimental paradigms, with particular focus on temporal dynamics and potential modulators of neural and clinical treatment responsivity. Within the predominantly obese and metabolically dysregulated populations investigated to date, the reviewed literature suggests that GLP-1RAs modulate distributed CNS systems, particularly involved in reward processing and appetitive control. Attenuation of reward-related responsivity following acute administration was among the more consistent findings across studies. Emerging evidence also suggests that GLP-1RAs differentially modulate reward-related processes rather than uniformly attenuate reward function, with neural effects further varying according to metabolic status and eating behavioral phenotype. Notably, several studies demonstrated more pronounced CNS modulation during early treatment phases, with diminished or altered effects following prolonged administration. However, the current neuroimaging literature is constrained by small sample sizes, substantial methodological heterogeneity, and sparse longitudinal evidence, restricting direct comparison across studies and limiting definitive conclusions regarding GLP-1RA-related CNS effects. Additionally, given the limited investigation of neurodegenerative and psychiatric populations, the generalizability of these findings to such clinical groups remains uncertain. Improved characterization of GLP-1RA-related CNS effects may ultimately help clarify putative mechanisms of action, identify clinically relevant sources of interindividual variability and treatment responsivity, and inform the potential therapeutic relevance of these agents across broader clinical populations.

## Supplementary Material

1

## Figures and Tables

**Fig. 1.**
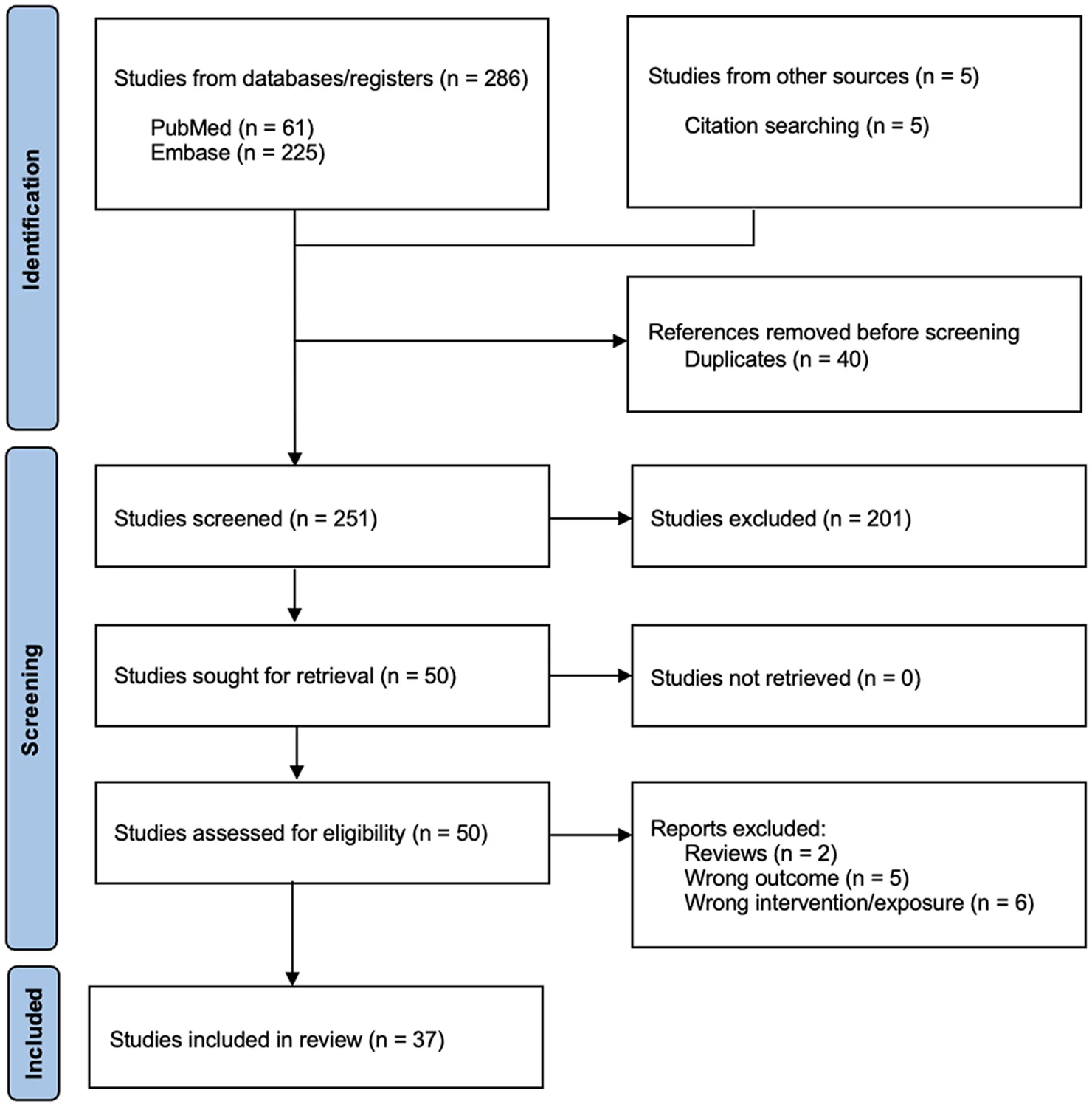
PRISMA Flow Diagram of systematic literature search.

**Fig. 2.**
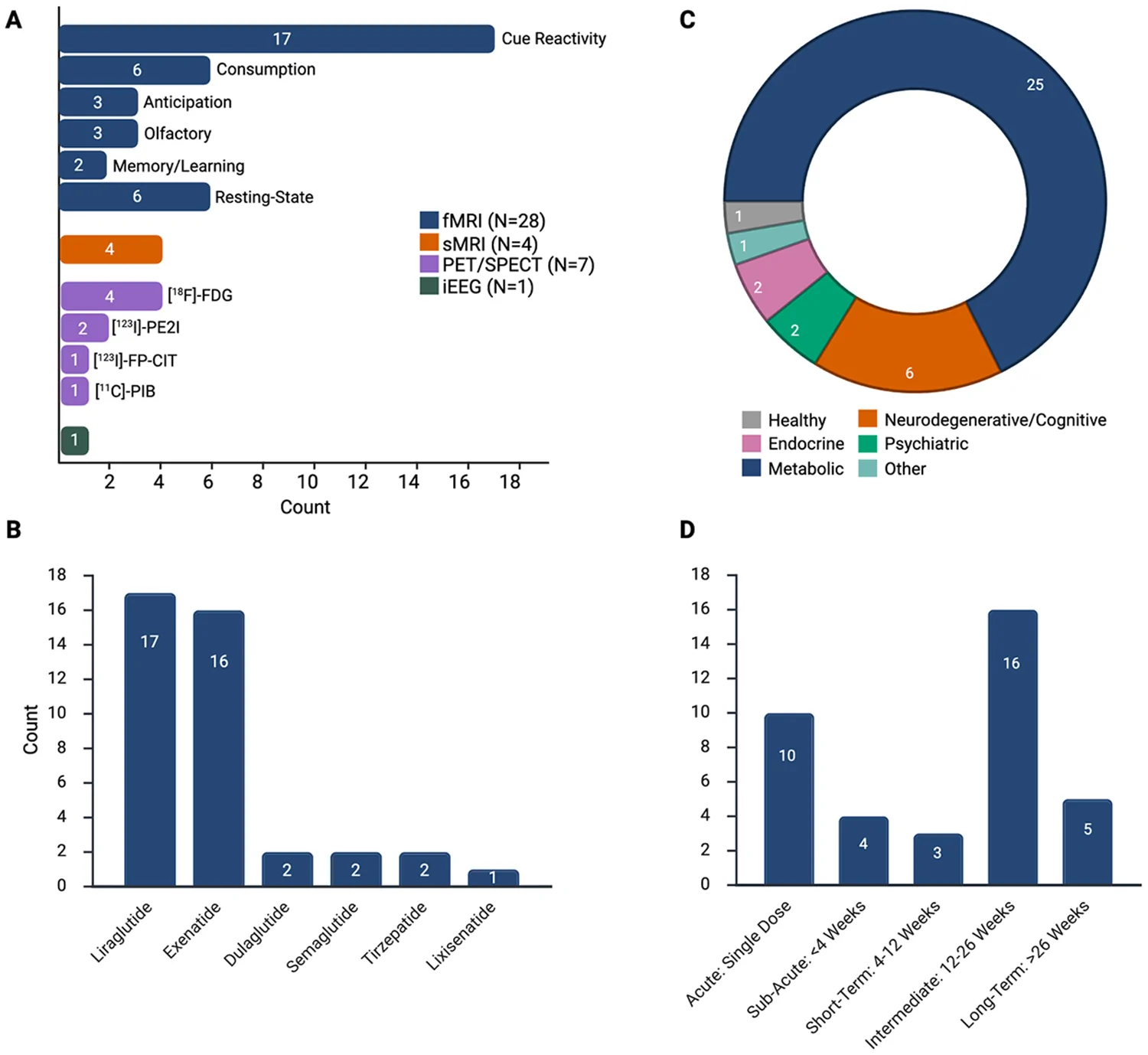
Study Characteristics. (A) Imaging modalities and experimental paradigms used across reviewed studies; (B) Distribution of GLP-1 receptor agonists (GLP-1RAs) investigated; (C) Clinical populations included; and (D) Duration of GLP-1RA treatment across studies. Category totals may exceed the total number of reviewed studies (N = 37) as some included multiple GLP-1RAs, imaging modalities, or experimental paradigms. Further details are provided in Supplementary Tables 1-5. Abbreviations: fMRI, functional magnetic resonance imaging; iEEG, intracranial electroencephalography; PET, positron emission tomography; sMRI, structural magnetic resonance imaging; SPECT, single-photon emission computed tomography. Created in BioRender. B, L. (2026) https://BioRender.com/zgr20es

**Fig. 3.**
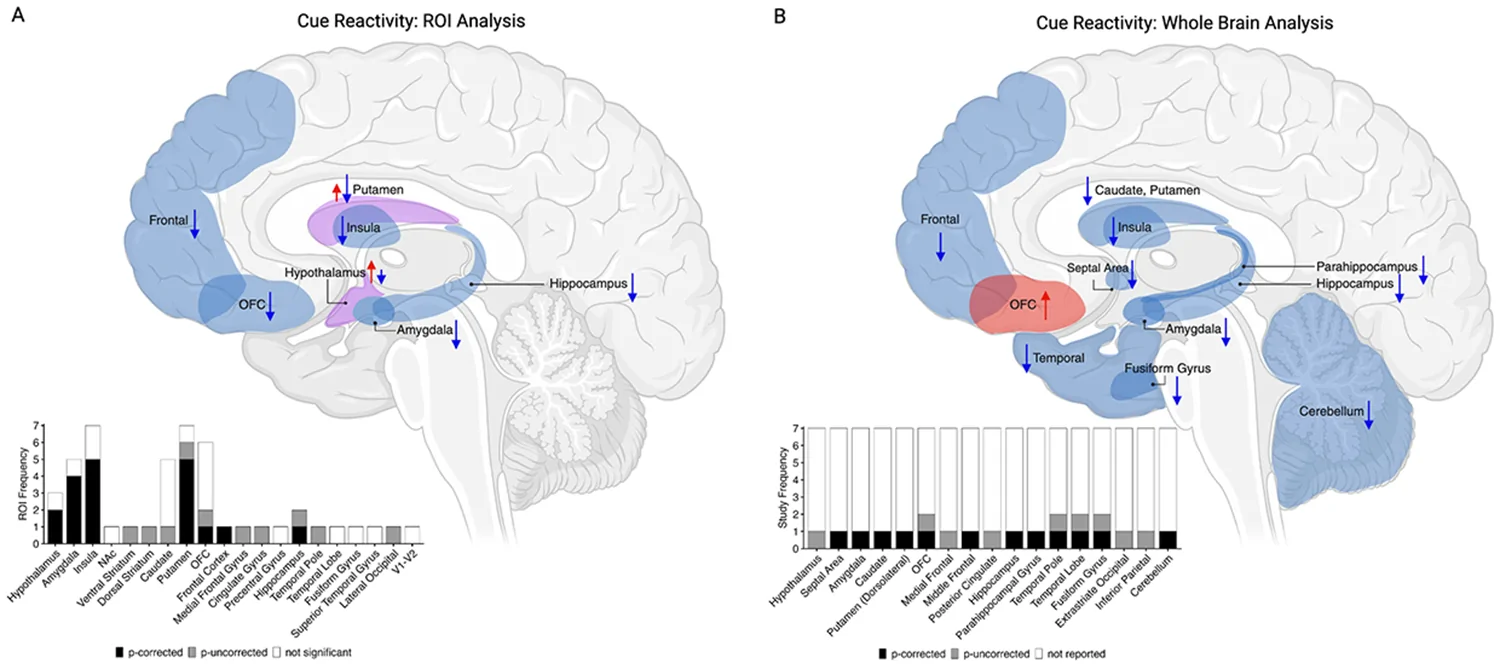
GLP-1RA-induced CNS effects during cue reactivity. **(A)** ROI analysis. Bar chart indicates the frequency with which individual regions were investigated as predefined ROIs across independent studies; Colors denote p-corrected, p-uncorrected, or non-significant findings. Brain visualization includes only p-corrected regions. **(B)** Whole-brain analysis. Bar chart indicates the total number of whole-brain studies (N = 7); Colors denote p-corrected, p-uncorrected, or unreported regions. Brain visualization includes only p-corrected regions. Visualized and tallied findings were limited to within-group, between-group, or treatment-related activation effects; Brain–behavior associations were excluded. Red indicates increased activation; Blue indicates decreased activation; Purple indicates both increased and decreased activation reported. Created in BioRender. B, L. (2026) https://BioRender.com/5sul1ra

**Fig. 4.**
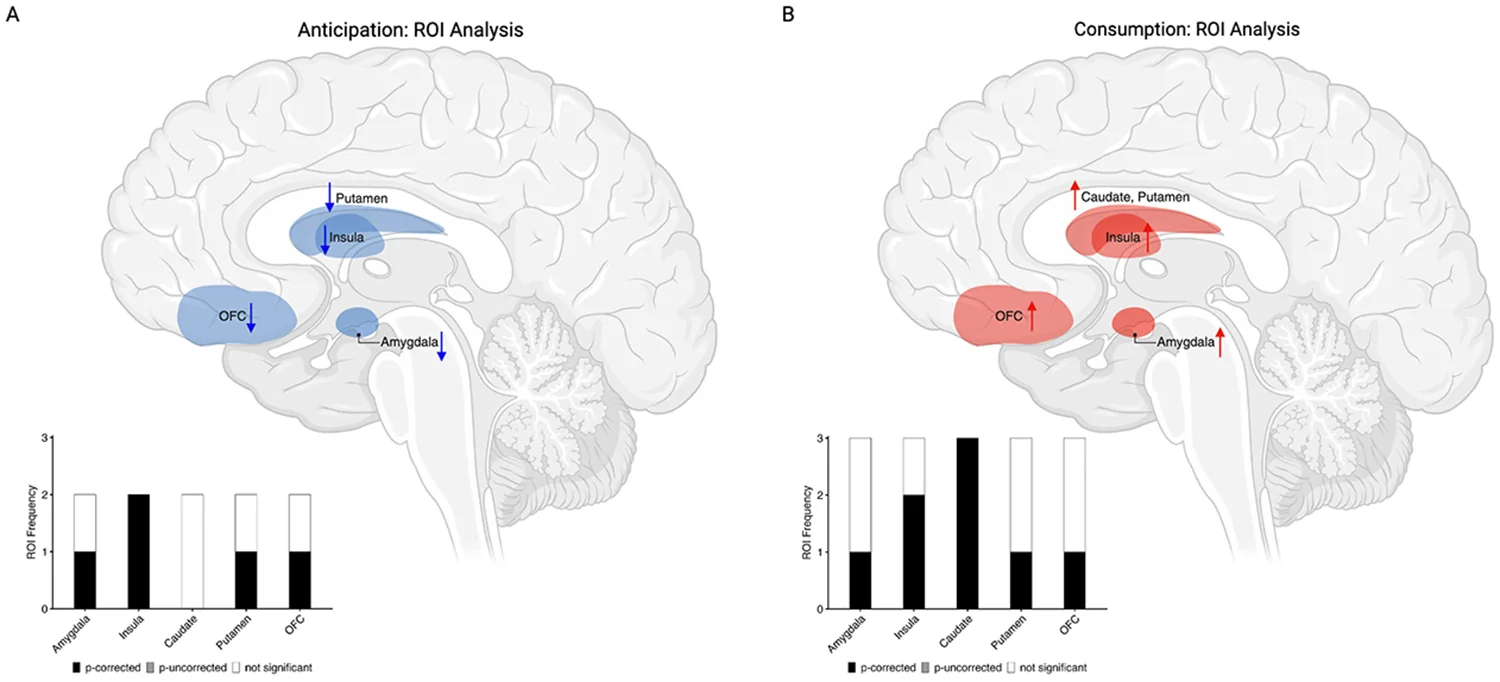
GLP-1RA-induced CNS effects during anticipation and consumption. Results of ROI analysis for (A) reward anticipation task, and (B) reward consumption task. Bar charts indicate the frequency with which individual regions were investigated as predefined ROIs across independent studies; Colors denote p-corrected, p-uncorrected, or non-significant findings. Brain visualization includes only p-corrected regions. Visualized and tallied findings were limited to within-group, between-group, or treatment-related activation effects; Brain–behavior associations were excluded. Red indicates increased activation; Blue indicates decreased activation. Created in BioRender. B, L. (2026)https://BioRender.com/vc291xe

**Fig. 5.**
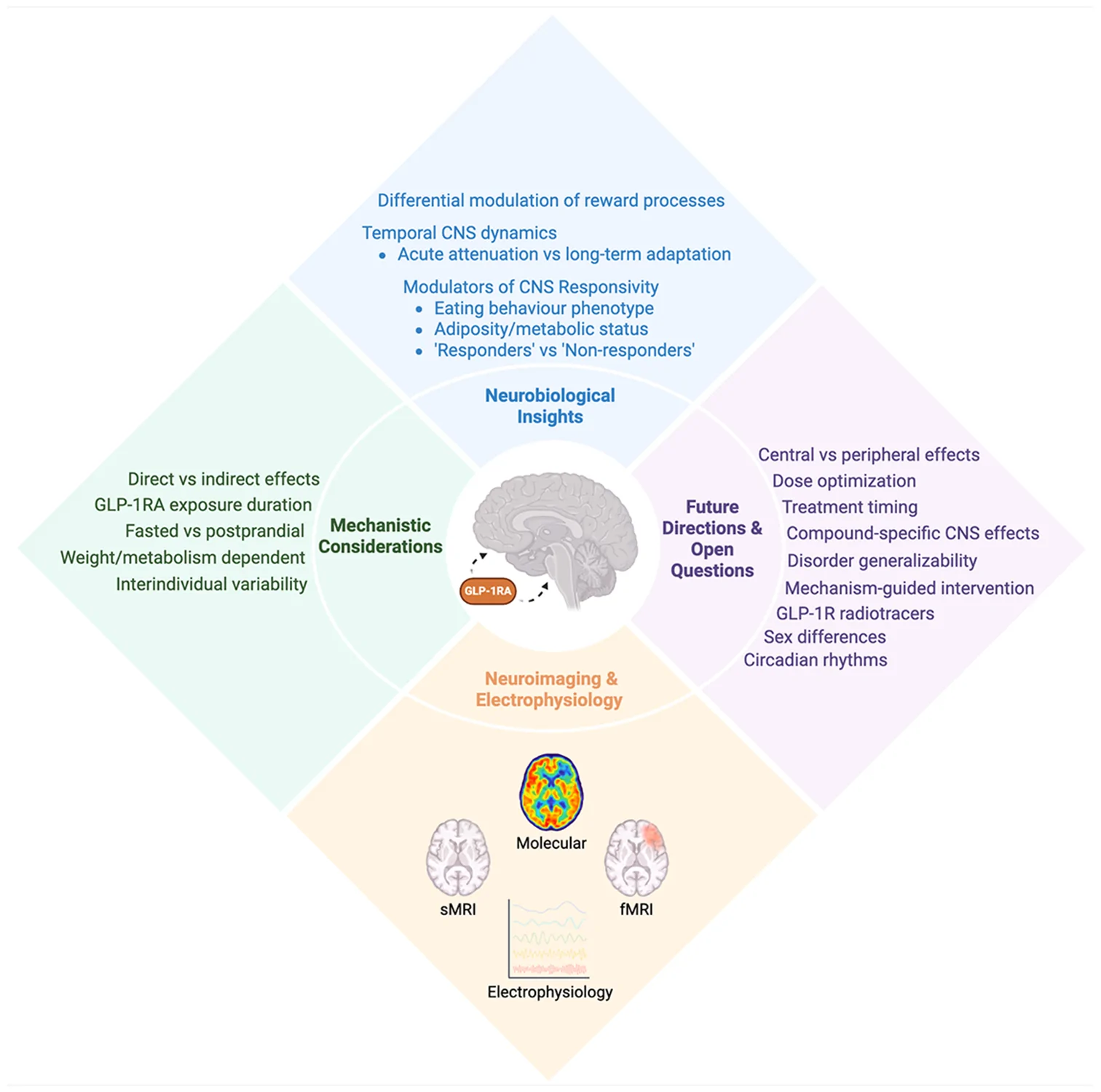
Overview of the emerging neurobiological insights and patterns identified across the reviewed clinical neuroimaging literature, mechanistic considerations influencing interpretation of GLP-1RA-induced CNS effects, and open questions and priorities for future research. Created in BioRender. B, L. (2026) https://BioRender.com/lkq1dpz

## Appendix A. Supporting information

Supplementary data associated with this article can be found in the online version at doi:10.1016/j.neubiorev.2026.106990.
