# Supplementary material for: Central nervous system effects of GLP-1 receptor agonists: A systematic review of human neuroimaging studies
Source: Neurosci Biobehav Rev. Author manuscript; Available in PMC 2026 Oct 29. (PMC13717676; doi:10.1016/j.neubiorev.2026.106990)
Supplement: Supplementary file 1 [file NIHMS2214437-supplement-1.docx]

**Central Nervous System Effects of GLP-1 Receptor Agonists: A Systematic Review of Human Neuroimaging Studies**

**Leyla R Brucar^1^, Kylee Miller^1^, Nora D Volkow^1^**

**^1^**National Institute on Alcohol Abuse and Alcoholism, Bethesda, Maryland

**Supplementary Materials**

**Supplementary Methods**

1. Search String

**Supplementary Tables**

1. **Table 1:** Functional MRI: Task Paradigms
2. **Table 2:** Functional MRI: Resting-State Paradigm
3. **Table 3:** Structural MRI
4. **Table 4:** Molecular Imaging
5. **Table 5:** Intracranial EEG
6. **Table 6:** Risk of Bias/Quality Assessment of Controlled Intervention Studies
7. **Table 7:** Risk of Bias/Quality Assessment of Pre-Post Studies
8. **Table 8**: Risk of Bias/Quality Assessment of Case Series Studies

**Supplementary Methods**

***Search String***

### (("GLP-1 receptor agonist*" OR "GLP 1 receptor agonist*" OR "GLP1 receptor agonist*" OR "glucagon-like peptide-1 receptor agonist*" OR "glucagon like peptide 1 receptor agonist*" OR "GLP-1 analogue*" OR "GLP 1 analogue*" OR "GLP1 analogue*" OR "glucagon-like peptide-1 analogue*" OR "dual GIP GLP-1 receptor agonist*" OR "dual GIP GLP 1 receptor agonist*" OR "dual GIP GLP1 receptor agonist*" OR liraglutide OR semaglutide OR exenatide OR dulaglutide OR lixisenatide OR tirzepatide) ) AND (neuroimaging OR fMRI OR EEG OR iEEG OR PET OR MRI OR MEG OR "Functional Magnetic Resonance Imaging") AND (brain OR neural OR CNS) AND (areas OR signal OR activation OR activity OR connectivity OR binding OR uptake OR respons* OR correlates) AND humans[Mesh]

**Table 1. Functional MRI: Task Paradigms**

| **fMRI: Cue Reactivity** | | | | | | | |
| --- | --- | --- | --- | --- | --- | --- | --- |
| **Author, Year** | **Study Type** | **Sample Description (Sample size; mean Age; mean BMI)** | **Intervention (GLP-1RA; Comparator; Duration; Dose)** | **Modality: Paradigm (Contrasts/Metric); Scan Timepoints; Scan Feeding State** | **Whole Brain/ROI** | **Imaging Outcomes** | **Behavioral/**  **Metabolic Outcomes** |
| Schlögl et al., (2013)^1^ | Randomized double-blind crossover | **Obese** (n=24; 29 years; BMI=37; F=0)  **Subgroup analysis:** ‘**Responders’** (individuals with ≥10% ↓ caloric intake post-exenatide) =13; ‘**Nonresponders’**=11 | Exenatide vs placebo; **Duration:** acute; **Dose:** IV infusion, 0.12 pmol/kg/min | fMRI: Food cue reactivity (food vs non-food; eigenvector centrality), **Scan Timepoints:** placebo, exenatide; **Feeding State:** fasted | Hypothalamus | **Exenatide vs Placebo:** **Responders:** ↑ hypothalamic eigenvector centrality (food cues); **Nonresponders:** NS effect;  **Interaction (drug x food vs nonfood):** Responders ↑ centrality (Talairach -3, -7, -14)  **Interaction (drug x responder/nonresponder group; caloric intake modulated):** significant (Talairach -3, -1, -17 (food vs nonfood)  **Covariate adjustment:** NR  **p-value correction:** SVC/FWE | **Responders:** ↓ caloric intake, hunger  **NS effects:** Nausea, buffet tastiness, food picture ratings, exenatide serum levels, blood glucose, neuropsychological and motor skills tasks |
| van Bloemendaal et al., (2014)^2^ | Randomized placebo-controlled crossover | **Obese-T2DM** (n=16; 61 years; BMI=34; F=8), **Normoglycemic-Obese** (n=16; 58 years; BMI=33; F=8), **Lean Controls** (n=16; 58 years; BMI=23; F=8) | Exenatide vs exenatide + exendin 9-39, placebo; **Duration:** acute; **Dose:** IV infusion, 50 ng/min for 30 min, then 25 ng/min | fMRI: Food cue reactivity (food vs non-food; high calorie vs non-food); **Scan Timepoints:** placebo, exenatide, exenatide + exendin 9-39; **Feeding State:** fasted, with somatostatin pancreatic-pituitary clamp | Insula, putamen, caudate, amygdala, OFC | **Within-Group (Exenatide vs Placebo):** **Normoglycemic-Obese:** ↓ R amygdala, insula (food cues); ↓ R insula, L OFC (high-calorie cues); **Obese-T2DM:** ↓ bilateral insula, L putamen, R OFC (food cues; high-calorie cues); **Lean:** NS effects;  **Exendin 9–39** **Pretreatment: Normoglycemic-Obese, Obese-T2DM:** largely blocked exenatide effects  **Associations (Δ exenatide vs placebo)**: Δ caloric intake +ve correlated with Δ CNS response (food cues) in **Obese-T2DM** (bilateral insula, R caudate), **Normoglycemic-Obese** (bilateral OFC, bilateral insula, R caudate); **Lean:** NS effects;  **Covariate adjustment:** NR  **p-value correction:** FWE | **Exenatide vs Placebo:** ↓ caloric intake (all groups); effect attenuated with exendin 9–39 (all groups)  **NS effects**: hunger, fullness, appetite, prospective food consumption, desire to eat, feelings of nausea. |
| ten Kulve et al., (2015)^3^ | Randomized crossover | **T2DM** (n=20; 59 years; BMI=32; F= NR) | Liraglutide vs insulin glargine; **Duration:** 12 weeks; **Dose:** 0.6–1.8 mg/day (weekly 0.6-mg escalation to max 1.8 mg) | fMRI: Food cue reactivity (food vs non-food; high calorie vs non-food); **Scan Timepoints:** baseline, 10-days, 12-weeks; **Feeding State:** fasted, postprandial | Insula, putamen, caudate, amygdala, OFC | **Liraglutide vs Insulin: Baseline:** NS effects; **10-days:** ↓ bilateral insula (fasted: high-calorie cues); ↓ L putamen, L amygdala (postprandial: high-calorie cues, food cues); ↑ meal-induced ↓ L putamen, L amygdala (fasted – postprandial: food cues); **12-weeks:** NS effects  **Covariate adjustment:** treatment order  **p-value correction:** SVC/FWE | **Liraglutide vs Insulin – 12-weeks:** ↓ weight, BMI, waist circumference, body fat, HbA1c, fasting glucose, total cholesterol, hunger scores  **NS effects:** heart rate, BP, ad libitum calorie intake |
| van Bloemendaal et al., (2015)^4^ | Randomized placebo-controlled crossover | **Obese-T2DM** (n=16; 61 years; BMI=34; F=8), **Normoglycemic-Obese** (n=16; 58 years; BMI=33; F=8), **Lean Controls** (n=16; 58 years; BMI=23; F=8) | Exenatide vs placebo; **Duration:** acute; **Dose:** IV infusion, 50 ng/min for 30 min, then 25 ng/min | fMRI: Food cue reactivity (food vs non-food); **Scan Timepoints:** placebo, exenatide; **Feeding State:** fasted, with somatostatin pancreatic-pituitary clamp | Insula, putamen, caudate, amygdala, OFC | **Normoglycemic-Obese - Emotional Eating Association: Placebo:** +ve correlated with R insula (food: high-calorie cues**); Exenatide:** +ve correlated with less ↓ bilateral amygdala activation (food cues)  **Obese-T2DM -** **Emotional Eating Association: Placebo:** +ve correlated with bilateral amygdala, bilateral inferior OFC, R insula (food cues), R inferior OFC (high calorie); **Exenatide:** +ve correlated with less ↓ R insula activation (food cues);  **Lean - Emotional Eating Association: Placebo**: +ve correlated with L insula (high-calorie cues)**; Exenatide:** NS effects;  **Covariate adjustment:** BMI  **p-value correction:** SVC/FWE | **Emotional Eating - Ad libitum intake Association:** NS effects (placebo/exenatide; all groups) |
| Eldor et al., (2016)^5^ | Experimental crossover intervention | **Obese** (n=10; 45 years; BMI=33; F=5), **Lean** (n=10; 44 years; BMI=24; F=6) | Exenatide vs saline; **Duration:** acute; **Dose:** IV infusion, 0.3 µg loading dose, then 0.05 µg/min | fMRI: Food cue reactivity (high calorie food vs non-food); **Scan Timepoints:** baseline, exenatide/saline; **Feeding State:** fasted; glycemic clamp during scans | Insula, amygdala, hypothalamus, hippocampus, frontal cortex | **Within-Group (Exenatide vs Saline):** **Obese:** ↑ hypothalamus (high-calorie cues), ↓ L insula, R amygdala, R hippocampus, frontal cortex (high-calorie cues); **Lean:** NS effects  **Saline - Obese vs Lean:** ↑ L insula, R amygdala, R hippocampus, frontal cortex (obese; high-calorie cues); ↑ hypothalamus (lean; high-calorie cues)  **Covariate adjustment:** NR  **p-value correction:** GRF | **Exenatide:** ↑ insulin secretion (lean > obese) |
| Farr et al., (2016)^6^ | Randomized double-blind placebo-controlled crossover | **T2DM** (n=20; 50 years; BMI=31; F=9*)* | Liraglutide vs placebo; **Duration:** 17 days; **Dose:** 0.6–1.8 mg/day (weekly 0.6-mg escalation to max 1.8 mg for final 3 days) | fMRI: Food cue reactivity (highly- vs less-desirable foods); **Scan Timepoints:** 17-day endpoints (placebo, liraglutide); **Feeding State:** fasted; postprandial | Whole brain | **Hormone-Food Cue Associations - Liraglutide vs Placebo (17 days):** **Fasted:** **GIP levels** -ve correlated with ↑ insula (p-uncorr); **leptin** +ve correlated with ↑ thalamus, parietal cortex, pre-SMA; **leptin** -ve correlated with ↓ DLPFC, midbrain, cerebellum (p-FWE); **Postprandial:** NS effects  **Covariate adjustment:** NR | **Liraglutide vs Placebo:** ↓ % Δ leptin; ↑ GIP; **Association:** -ve correlation between % Δ leptin and fullness; |
| Farr et al., (2016)^7^ | Randomized double-blind placebo-controlled crossover | **T2DM** (n=18; 49 years; BMI=31; F=9) | Liraglutide vs placebo; **Duration:** 17 days; **Dose:** 0.6–1.8 mg/day (weekly 0.6-mg escalation to max 1.8 mg for final 3 days) | fMRI: Food cue reactivity (highly vs less desirable foods; all-food vs non-food); **Scan Timepoints:** 17-day endpoints (placebo, liraglutide); **Feeding State:** fasted | Whole brain;  Secondary ROI Analysis: Insula, putamen, OFC, hypothalamus; | **Liraglutide vs Placebo (17 days, Fasted):** **Whole Brain Analysis:** ↓ inferior parietal cortex (highly vs less desirable; p-uncorrected); **ROI Analysis:** ↓ insula, putamen (highly vs less desirable; p-SVC); NS effects: Hypothalamus  **Food Cue Associations - Liraglutide vs Placebo (17 days, Fasted)**: ↓ eating pleasantness correlated with ↓ inferior parietal; hunger/appetite +ve correlated with ↑ parietal, precuneus, cuneus, occipital (p-uncorrected); nausea -ve correlated with R cingulate gyrus (p-FWE)  **Covariate adjustment:** NR  **p-value correction:** Whole Brain (uncorrected); ROI analysis: SVC | **Liraglutide vs Placebo:** ↓ energy intake, fasting blood glucose**;** ↑ fullness (fasted)  **NS effects**: fructosamine, HbA1c, lipid profiles, BP, BMI, fat mass, neurocognitive testing (CANTAB)  **Covariate adjustment:** baseline values |
| Bae et al., (2019)^8^ | Randomized single-blind crossover | **T2DM**: **Lean** (n=15; 62 years; BMI=22; F=11), **Obese** (n=14; 59 years; BMI=28; F=7);  **Subgroup analysis** (‘**Responders’**: ↓ ad libitum caloric intake post-Lixisenatide): n=17 | Lixisenatide vs placebo; **Duration:** acute; **Dose:** 10 µg | fMRI: Food cue reactivity (food vs non-food; high-calorie vs low-calorie); **Scan Timepoints:** placebo, lixisenatide; **Feeding State:** fasted | Whole brain | **Obese-T2DM vs Lean-T2DM:** **Placebo:** ↑ hypothalamus, pineal gland, parietal cortex (high-calorie cues); ↑ OFC (high-calorie cues); ↑ visual cortex (food cues); **Lixisenatide:** ↓ fusiform gyrus (high-calorie cues);  **Food Cue Associations (Lixisenatide):** Baseline fullness, post-fMRI satiety, post-fMRI fullness +ve correlated with baseline hypothalamus, pineal gland (high calorie cues); post-buffet satiety, fullness, post-fMRI satiety, craving inversely correlated with visual cortex (food cues);  **Subgroup Analysis (‘Responders’) – Group x Treatment Interaction:** posterior cingulate, medial frontal activation ↓ obese, ↑ lean (high-calorie cues); hypothalamus, temporal lobe, OFC activation ↓ obese, ↑ lean in (food cues);  **Food Cue Associations (Lixisenatide; ‘Responders’ Subgroup Analysis)**: Baseline craving, post-fMRI hunger +ve correlated with posterior cingulate, medial frontal (high-calorie cues);  **Covariate adjustment:** NR  **p-value correction:** uncorrected | **Lixisenatide (all groups):** ↓ caloric intake in 17/29 participants (8 lean, 9 obese); **NS effects:** Δ caloric intake between obese-T2DM vs lean-T2DM |
| Binda et al., (2019)^9^ | Within-subject controlled intervention, non-randomized | **Obese** (n=10; 45 years; BMI=33; F=5); **Lean** (n=10; 44 years; BMI=24; F=6), | Exenatide vs saline; **Duration:** acute; **Dose:** IV infusion, 0.3 µg loading dose, then 0.05 µg/min | fMRI: Food cue reactivity (food vs non-food; % raw signal intensity change); **Scan Timepoints:** saline, exenatide**; Feeding State:** fasted | Whole-cortical surface  ROIs: V1-V2, lateral occipital, fusiform gyrus, temporal pole | **Whole-cortical surface analysis:** **Drug × Group Interaction:** NS effects;  **Exenatide vs Placebo** **(Drug Main Effect):** ↓ temporal pole; ↑ extrastriate occipital (all cues). **Food vs Non-Food** **(Cue Main Effect):** ↑ fusiform, temporal, limbic (food cues); ↑ V1–V2 (non-food cues) **Obesity vs Lean** **(Group Main Effect):** NS effect;  **ROI analysis:**  **Exenatide vs Placebo** **(Drug Main Effect):** ↑ lateral occipital (all cues)  **Food vs Non-Food** **(Cue Main Effect):** ↑ fusiform gyrus (food > non-food)  **Obesity vs Lean** **(Group Main Effect):** NS effects  **Drug x Cue Interaction**: ↑ temporal pole (food cues);  **Covariate adjustment:** NR  **p-value correction:** uncorrected | NR |
| Farr et al., (2019)^10^ | Randomized double-blind placebo-controlled crossover | **Obese** (n=20; 55 years; BMI=35; F=9) | Liraglutide vs placebo; **Duration:** 5 weeks; **Dose:** 0.6-3.0 mg/day (weekly 0.6-mg escalation to max 3.0 mg) | fMRI: Food cue reactivity (highly- vs less-desirable food; food vs non-food); **Scan** **Timepoints:** 5-week endpoints **(**placebo, liraglutide)**; Feeding State:** fasted | Whole brain | **Liraglutide vs Placebo – 5 weeks:** **No covariate adjustment:** NS effects (food cues); **With BMI/weight covariate adjustment:** ↑ R OFC (food cues)  **p-value correction:** FWE | **Liraglutide vs Placebo:** ↓ weight, BMI, waist circumference, fasting glucose, cholesterol; ↑ stop-signal performance; ↓ perceived food intake, eating pleasantness; Δ weight +ve correlated with food intake & eating pleasantness**; NS effects:** nausea, CANTAB |
| van Ruiten et al., (2022)^11^ | Secondary analysis of randomized double-blind, placebo-controlled | **Obese-T2DM: Exenatide** (n=17; 65 years; BMI=33; F=6), **Placebo** (n=16; 61 years; BMI=32; F=4),  **Exenatide + Dapagliflozin** (n=16; 64 years; BMI=31; F=4), **Dapagliflozin** (n=16; 64 years; BMI=32; F=4) | Exenatide vs placebo, dapagliflozin, exenatide + dapagliflozin; **Duration:** 16 weeks; **Dose:** 5-10 µg twice daily (increased to max 10 µg after 4 weeks) | fMRI: Food cue reactivity (low calorie vs non-food); **Scan Timepoints:** baseline, 10 days, 16 weeks**; Feeding State:** fasted | Insula, caudate, putamen, amygdala, OFC | **Exenatide vs Placebo: 10 days:** ↑ R putamen (low-calorie cues); **16 weeks:** NS effects  **Covariate adjustment:** NR  **p-value correction:** SVC/FWE | **Exenatide – 16 weeks:** ↓ body weight, body fat, waist circumference  **NS effects**: Δ in ad libitum caloric intake |
| van Ruiten et al., (2022)^12^ | Secondary analysis of randomized open-label crossover | **Obese-T2DM** (n=20; 59 years; BMI=32; F=NR) | Liraglutide vs insulin glargine; **Duration:** 12 weeks; **Dose:** 0.6-1.8 mg/day (weekly 0.6-mg escalation to max 1.8 mg) | fMRI: Food cue reactivity (food vs non-food; high-calorie vs non-food); **Scan Timepoints:** baseline, 10 days, 12 weeks**; Feeding State:** fasted, postprandial | Insula, caudate, putamen, amygdala, OFC | **Emotional Eating Associations – Liraglutide vs Insulin: Baseline:** NS effects; **10 days:** +ve correlated with less ↓ activation (fasted: L amygdala, L caudate [food cues], L amygdala, R insula [high-calorie cues]; postprandial: R OFC, L insula [food cues]); **12 weeks:** NS effects;  **External Eating Associations – Liraglutide vs Insulin: Baseline:** +ve correlated with bilateral insula (food, high-calorie cues); **10 days:** +ve correlated with less ↓ activation (fasted: R insula [food cues]; postprandial: R insula, bilateral amygdala [food, high-calorie cues]); **12 weeks:** NS effects;  **Restrained Eating Associations – Liraglutide vs Insulin: Baseline:** NS effects**; 10 days:** NS effects**; 12 weeks:** +ve correlated with greater ↓ activation (fasted: bilateral insula [food cues]), L insula, right caudate [high-calorie cues])  **Covariate adjustment:** NR  **p-value correction:** SVC/FWE | **Emotional Eating, External Eating:** NS association with Δ in BMI, weight, body fat, calorie intake  **Restrained Eating:** -ve correlation with baseline BMI, weight, caloric intake  **Liraglutide – 12 weeks:** ↓ body weight  **NS effects:** Nausea |
| van Ruiten et al., (2022)^13^ | Randomized double-blind placebo-controlled | **Obese-T2DM**: **Exenatide** (n=17; 65 years; BMI=33; F=6), **Placebo** (n=16; 61 years; BMI=32; F=4);  **Dapagliflozin** (n=16; 64 years; BMI=32; F=4);  **Exenatide + Dapagliflozin** (n=16; 64 years; BMI=31; F=4) | Exenatide vs placebo, dapagliflozin, exenatide + dapagliflozin; **Duration:** 16 weeks; **Dose:** 5-10 µg twice daily (increased to max 10 µg after 4 weeks) | fMRI: Food cue reactivity (food vs non-food; high-calorie vs non-food); **Scan Timepoints:** baseline, 10 days, 16 weeks**; Feeding State:** fasted | Insula, caudate, putamen, amygdala, OFC | **Exenatide vs Placebo: 10 days:** ↓ L putamen (high-calorie cues)**; 16 weeks:** ↓ bilateral insula (high-calorie cues); ↓ R insula (food cues)  **Associations (**Δ CNS-Δ Clinical Parameters)**:** NS effects  **Covariate adjustment:** NR  **p-value correction:** SVC/FWE | **Exenatide – 16 weeks:** ↓ body weight, body fat, waist circumference;  **NS effects**: Δ in ad libitum caloric intake; appetite-related scores |
| Jensterle et al., (2025)^14^ | Randomized single-blind placebo-controlled | **PCOS** (n=30; 34 years; BMI=36; F=30); **Semaglutide**=15; **Placebo**=15 | Semaglutide vs placebo; **Duration:** 16 weeks; **Dose:** 0.25-1.0 mg/week (0.25 and 0.5 mg for 2 weeks each, then to max 1.0 mg) | fMRI: Food cue reactivity (calorie dense sweet/savory cues); **Scan Timepoints:** baseline, 16 weeks; **Feeding State:** fasted, postprandial | Whole brain | **Semaglutide vs Placebo – 16 weeks:** ↓ dorsolateral putamen (postprandial: calorie-dense cues)  **Covariate adjustment:** NR  **p-value correction:** clusterwise | **Semaglutide vs Placebo – 16-weeks:** ↓ body weight, BMI, HbA1c, HDL, waist circumference, fasting glucose; ↑ HR, taste recognition sensitivity, taste-related tongue gene transcription;  **Covariate adjustment:** baseline weight |
| Martin et al., (2025)^15^ | Randomized partially blind  placebo-controlled parallel-group | **Obese/overweight:** **Tirzepatide** (n=37; 45 years; BMI=36; F=36), **Liraglutide** (n=38; 44 years; BMI=36; F=25), **Placebo** (n=39; 46 years; BMI=36; F=36);  **fMRI Sample:** **Baseline:** **Tirzepatide**=31, **Liraglutide**=34, **Placebo**=31 | Tirzepatide vs liraglutide, placebo; **Duration:** 6 weeks; **Dose:** Tirzepatide: 5-10 mg/week (increased to 10 mg after 3 weeks), Liraglutide: 0.6-3.0 mg/day (weekly 0.6-mg escalation to max 3.0 mg) | fMRI: Food cue reactivity (high palatable vs non-food; high fat/sugar vs non-food); **Scan Timepoints:** baseline**,** 3 weeks, 6 weeks**; Feeding State:** fasted | Insula, medial frontal gyrus, superior temporal gyrus, precentral gyrus, cingulate gyrus, hippocampus, putamen, OFC | **Tirzepatide vs Placebo (Δ Baseline): 3 weeks:** ↓ medial frontal gyrus, cingulate, hippocampus, OFC (high-fat/sugar cues); **6 weeks:** NS effects  **Liraglutide vs Placebo (Δ Baseline): 3 weeks/6 weeks:** NS effect  **Tirzepatide vs Liraglutide (Δ Baseline): 3 weeks:** NS effects; **6 weeks:** ↓ medial frontal gyrus, cingulate, OFC (high-fat/sugar cues);  **Covariate adjustment:** baseline values**,** scanner ID  **p-value correction:** uncorrected | **Body weight, energy intake (****3 weeks, 6 weeks):** Tirzepatide ↓ vs liraglutide/placebo; liraglutide ↓ vs placebo;    **Impulsivity (BIS; 3 weeks, 6 weeks):** Tirzepatide ↓ vs placebo;    **Eating behavior (EI; 3 weeks, 6 weeks):** **Cognitive restraint:** NS; **Disinhibition:** Tirzepatide ↓ vs liraglutide/placebo; **Hunger:** Tirzepatide ↓ vs liraglutide/placebo; liraglutide ↓ vs placebo (6-weeks only);    **Food craving (FCI, FCQ-S; 3-weeks, 6-weeks):** Tirzepatide ↓ vs liraglutide/placebo; liraglutide ↓ vs placebo (3-weeks only);  **p-value correction:** uncorrected |
| Winzeler et al., (2021)^16^ | Exploratory fMRI substudy of randomized double-blind placebo-controlled crossover (with matched controls) | **Primary Polydipsia** (n=15; 32 years; BMI=NR, F=11), **Controls** (n=15; 29 years; BMI=NR; F=12)  **Subgroup Analysis** (‘High Thirst’ state): Placebo=14; Controls=10 | Dulaglutide vs placebo, controls; **Duration:** 3 weeks; **Dose:** 1.5 mg/week | fMRI: Beverage cue reactivity (beverage vs chair cues); **Scan Timepoints**: baseline, 3-week endpoints (placebo, dulaglutide); **Feeding State:** Fasted | Whole Brain | **Primary Polydipsia (Treatment x Stimulus Interaction; Main Effects):** NS effects;  **Placebo vs Controls: Group × Stimuli Interaction:** NS effects;  **High-Thirst Subgroup:** NS effects  **Covariate adjustment:** NR  **p-value correction:** cluster-level | **Dulaglutide vs Placebo** – 3 weeks: ↓ fluid intake, urine output, thirst ratings; ↑ quality of life;  **Covariate adjustment:** baseline measurement |
| Klausen et al., (2022)^17^ | Randomized double-blind placebo-controlled | **AUD: Exenatide** (n=28; 51 years; BMI=NR, F=12), **Placebo** (n=33; 49 years; BMI=NR; 13); **Controls** (n=25; 49 years; BMI=NR; F=10;)  **fMRI Completers:** Exenatide=17, Placebo=16,  Controls=25 | Exenatide vs placebo, controls; **Duration:** 26 weeks; **Dose:** 2 mg/week | fMRI: Alcohol cue reactivity (alcohol vs neutral cues); **Scan Timepoints:** baseline, 26 weeks; **Feeding state:** NR | ROIs: dorsal striatum, ventral striatum, NAc, putamen, caudate; Exploratory:  Whole brain | **ROI Analysis:**  **Treatment x Time Interaction:** ↓ ventral/dorsal striatum, putamen;  **Between-Groups (Exenatide vs Placebo): Baseline:** NS effects; **26 weeks:** ↓ ventral striatum  **Within-Group (Baseline vs 26 weeks):** **Exenatide:** ↓ ventral/dorsal striatum; **Placebo:** NS effects;  **Whole-Brain Analysis:**  **Between-Group (Exenatide vs Placebo): Baseline:** NS effects; **26 weeks:** ↓ L caudate, septal area, R middle frontal gyrus (alcohol cues)  **Within-Group (Baseline vs 26 weeks):** **Exenatide:** ↓ L temporal lobe (middle/superior temporal gyrus), R temporal lobe (middle temporal gyrus, temporal pole: middle temporal gyrus, superior temporal gyrus); R Limbic (hippocampus, parahippocampal gyrus, amygdala, fusiform gyrus); L parahippocampal gyrus, hippocampus, amygdala, middle temporal gyrus, temporal pole: superior temporal gyrus, cerebellum, fusiform gyrus); **Placebo:** NS effects;  **All-AUD vs Controls: Baseline:** ↑ L superior/middle frontal gyrus, caudate, insula (alcohol cues); **26 weeks:** NS effects  **Covariate adjustment:** NR  **p-value correction:** ROI: uncorrected; Whole-brain: FWE/clusterwise/voxelwise | **Exenatide vs Placebo – 26 weeks:** ↓ BMI, HbA1c; ↑ urinary oxidative stress markers; NS effect: drinking-days  **Craving:** **Baseline:** all-AUD vs Control: ↑ craving; **26 weeks:** NS effects  **BMI Subgroup Analysis:** **BMI>30:** Exenatide ↓ heavy drinking (−23.6%), ↓ total intake; **BMI<25:** Exenatide ↑ heavy drinking days (+27.5%). |
| **fMRI: Anticipation and Consumption Tasks** | | | | | | | |
| **Author, Year** | **Study Type** | **Sample Description (Sample size; mean Age; mean BMI)** | **Intervention (GLP-1RA; Comparator; Duration; Dose)** | **Modality: Paradigm (Contrasts/Metric); Scan Timepoints; Scan Feeding State** | **Whole Brain/ROI** | **Imaging Outcomes** | **Behavioral/Metabolic Outcomes** |
| van Bloemendaal et al., (2015)^18^ | Randomized placebo-controlled crossover | **Obese-T2DM** (n=16; 61 years; BMI=34; F=8), **Normoglycemic-Obese** (n=15; 58 years; BMI=33; F=7), **Lean** (n=16; 58 years; BMI=23; F=8) | Exenatide vs exenatide + exendin 9-39, placebo; **Duration:** acute; **Dose:** IV infusion, 50 ng/min for 30 min, then 25 ng/min | fMRI: chocolate milk anticipation/consumption task (chocolate milk vs tasteless solution); **Scan Timepoints:** placebo, exenatide, exenatide + exendin 9-39; **Feeding State:** fasted, with somatostatin pancreatic-pituitary clamp | Insula, putamen, caudate, amygdala, OFC | **Within-Group (Exenatide vs placebo): Normoglycemic-Obese:** NS effects (anticipation); ↑ R OFC (consumption); **Obese-T2DM:** ↓ bilateral putamen, L insula, L amygdala (anticipation); ↑ L insula, bilateral putamen, L amygdala (consumption); **Lean:** ↓ bilateral OFC (anticipation); ↑ R caudate (consumption);  **Between-Groups:** NS effects (anticipation, consumption)  **Exendin 9–39 Pretreatment:** largely blocked CNS effects  **Associations:** BMI +ve correlated with R putamen (anticipation); BMI -ve correlated with R putamen (consumption);  **Covariate adjustment:** NR  **p-value correction:** SVC/FWE | **Exenatide (all groups):** ↓ caloric intake  **NS effects:** hunger, fullness, appetite, prospective food consumption, desire to eat, nausea |
| ten Kulve et al., (2016)^19^ | Randomized crossover | **Obese-T2DM** (n=20; 60 years; BMI=32; F=9) | Liraglutide vs insulin glargine; **Duration:** 12 weeks; **Dose:** 0.6-1.8 mg/day (weekly 0.6-mg escalation to max 1.8 mg) | fMRI: chocolate milk consumption task (chocolate milk vs baseline; chocolate milk vs tasteless solution); **Scan Timepoints:** baseline**,** 10 days, 12 weeks; **Feeding State:** fasted | Insula, caudate, putamen, amygdala, OFC | **Liraglutide vs Insulin: Baseline:** NS effects; **10 days:** ↑ R caudate, R insula (chocolate milk vs baseline)**; 12 weeks:** NS effects; **Insulin vs Liraglutide: 12-weeks:** ↑ R amygdala  **Associations – Liraglutide vs Insulin:** ↑ Δ in R insula activation (10 days) +ve correlated with ↑ Δ in weight (12 weeks)  **Covariate adjustment:** NR  **p-value correction:** SVC/FWE | **Liraglutide vs insulin glargine (Δ Baseline): 10 days:** ↓ fasting glucose; ↑ heart rate; **12-weeks:** ↓ weight, BMI, waist circumference, HbA1c, fasting glucose |
| van Ruiten et al., (2022)^12^ | Secondary analysis of randomized open-label crossover | **Obese-T2DM** (n=20; 59 years; BMI=32; F=NR) | Liraglutide vs insulin glargine; **Duration:** 12 weeks; **Dose:** 0.6-1.8 mg/day (weekly 0.6-mg escalation to max 1.8 mg) | fMRI: chocolate milk anticipation/consumption task (chocolate milk vs tasteless solution); **Scan Timepoints:** baseline, 10 days, 12 weeks; **Feeding State:** fasted | Insula, caudate, putamen, amygdala, OFC | **Emotional Eating Associations – Liraglutide vs Insulin: Baseline:** +ve correlated with bilateral insula (anticipation); -ve correlated with bilateral insula (consumption); **10 days/12 weeks:** NS effects;  **External Eating Associations – Liraglutide vs Insulin: Baseline:** -ve correlated with R insula (consumption); **10 days/12 weeks:** NS effects  **Restraint Eating Association – Liraglutide vs Insulin: Baseline:** -ve correlated with R amygdala (anticipation)**;** +ve correlated with R amygdala (consumption); **10 days:** NS effects**; 12 weeks:** +ve correlated with greater ↓ bilateral caudate (anticipation);  **Covariate adjustment:** NR  **p-value correction:** SVC/FWE | **Emotional Eating, External Eating:** NS associations with Δ in BMI, weight, body fat, calorie intake  **Restrained Eating:**  -ve correlated with baseline BMI, weight, caloric intake  **Liraglutide -12 weeks:** ↓ body weight, HbA1c, % body fat  **NS effects:** Nausea |
| van Ruiten et al., (2022)^20^ | Randomized double-blind placebo-controlled | **Obese-T2DM:** **Exenatide** (n=17; 65 years; BMI=33; F=6), **Placebo** (n=15; 61 years; BMI=32; F=4);  **Dapagliflozin** (n=16; 64 years; BMI=32; F=4), **Dapagliflozin + Exenatide** (n=15; 64 years; BMI=31; F=4), | Exenatide vs placebo, dapagliflozin, exenatide + dapagliflozin; **Duration:** 16 weeks; **Dose:** 5-10 µg twice daily (increased to max 10 µg after 4 weeks**)** | fMRI: chocolate milk anticipation/consumption task (chocolate milk vs tasteless solution); **Scan Timepoints:** baseline, 10 days, 16 weeks; **Feeding State:** fasted | Insula, caudate, putamen, amygdala, OFC | **Exenatide vs placebo: 10 days:** NS effects; **16 weeks:** ↓ R insula (anticipation); ↑ L caudate (consumption);  **Associations (**Δ CNS-Δ Clinical Parameters)**:** NS effects  **Covariate adjustment:** NR  **p-value correction:** SVC/FWE | **Exenatide – 16 weeks:** ↓ body weight, body fat, waist circumference;  **NS effects**: Δ in ad libitum caloric intake; appetite-related scores |
| Coppin et al., (2023)^21^ | Randomized double-blind placebo-controlled parallel group | **Obese: Liraglutide** (n=20; 37 years; BMI=36; F=6), **Placebo** (n=24; 40 years; BMI=35; F=8) | Liraglutide vs placebo; **Duration:** 16 weeks; **Dose:** 0.6-3.0 mg/day (weekly 0.6-mg escalation to max 3.0 mg) | fMRI: milkshake consumption task (milkshake vs tasteless solution); **Scan Timepoints:** baseline, 16 weeks; **Feeding State:** fasted | Whole brain | **Liraglutide vs Placebo** (**Intervention x Timepoint Interaction):** NS effects  **Pleasantness/Liking (Main Effect):** ↑ vmPFC, bilateral amygdala correlated with milkshake liking (consumption; both groups)  **Covariate adjustment:** NR  **p-value correction:** FDR | **Liraglutide vs placebo – 16 weeks:** ↓ body weight;  **NS effect**: intervention on pleasantness/liking  **Covariate adjustment:** age, sex |
| Jensterle et al., (2025)^14^ | Randomized single-blind placebo-controlled | **PCOS:** (n=30; 34 years; BMI=36; F=30); **Semaglutide**=15, **Placebo**=15 | Semaglutide vs placebo; **Duration:** 16 weeks; **Dose:** 0.25-1.0 mg/week (0.25 and 0.5 mg for 2 weeks each, then to max 1.0 mg) | fMRI: taste task (glucose vs water); **Scan Timepoints:** baseline, 16 weeks; **Feeding State:** fasted, postprandial | Whole brain | **Semaglutide vs Placebo – 16 weeks:** ↑ angular gyrus (postprandial: sweet vs water); Fasted: NS effects  **Covariate adjustment:** NR  **p-value correction:** clusterwise | **Semaglutide vs Placebo – 16 weeks:** ↓ body weight, BMI, HbA1c, HDL, waist circumference, fasting glucose; ↑ HR, ↑ taste recognition sensitivity, ↑ taste-related tongue gene transcription;  **Covariate adjustment:** baseline weight |
| **fMRI: Olfactory Tasks** | | | | | | | |
| **Author, Year** | **Study Type** | **Sample Description (Sample size; mean Age; mean BMI)** | **Intervention (GLP-1RA; Comparator; Duration; Dose)** | **Modality: Paradigm (Contrasts/Metric); Scan Timepoints; Scan Feeding State** | **Whole Brain/ROI** | **Imaging Outcomes** | **Behavioral/**  **Metabolic Outcomes** |
| Zhang et al., (2019)^22^ | Randomized parallel-group intervention | **Obese-T2DM:** **Liraglutide** (n=9; 52 years; BMI=32 F=3),  **Exenatide** (n=10; 53 years; BMI=32; F=4) | Liraglutide vs exenatide; **Duration:** 3 months; **Dose:** liraglutide: 0.6-1.8 mg/day (increased to max 1.8 mg over 2 weeks), exenatide: 5-10 µg twice daily (increased to max 10 µg over 2 weeks) | fMRI: odor task (fresh air vs rest; lavender scent vs rest); **Scan** **Timepoints:** baseline, 3 months; **Feeding State:** NR | ‘Olfactory network’ ROIs: Parahippocampus, amygdala, piriform cortex, insula, OFC, hippocampus, entorhinal cortices | **Liraglutide/Exenatide vs Baseline:** ↑ R Parahippocampus (scent vs rest); **With additional HbA1c/BMI covariate adjustment:** NS effects  **Liraglutide vs Exenatide: 3 months:** NS effects  **Covariate adjustment:** age, sex, education, blood pressure, serum lipids  **p-value correction:** GRF | **Liraglutide/Exenatide vs Baseline:** ↓ weight, BMI, HbA1c, systolic BP; ↑ 2-hour postprandial insulin, C-peptide, HDL;  **Cognition/Olfaction - Liraglutide/Exenatide vs Baseline:** ↑ memory, general cognition, olfactory identification (**covariate adjustment:** age, sex, education, duration, BP); **With additional HbA1c/BMI adjustment:** NS effects;  **Liraglutide vs Exenatide: 3 months:** NS effects |
| Cheng et al., (2022)^23^ | Randomized parallel-group open-label | **T2DM: Liraglutide** (n=12; 52 years; BMI=27; F=6);  **Dapagliflozin** (n=12; 57 years; BMI=27; F=5), **Acarbose** (n=12; 56 years; BMI=27; F=5) | Liraglutide vs dapagliflozin, oral acarbose; **Duration:** 16 weeks; **Dose:** 0.6-1.8 mg/day (increased to max 1.8 mg over 2 weeks) | fMRI: odor task (fresh air vs rest; lavender scent vs rest); **Scan Timepoints:** baseline, 16 weeks; **Feeding State:** NR | ‘Olfactory network’ ROIs: Parahippocampus, amygdala, piriform cortex, insula, OFC, hippocampus, entorhinal cortices | **Liraglutide vs Baseline:** ↑ L hippocampus (scent vs rest)  **Covariate adjustment:** NR  **p-value correction:** GRF  **SEM Analysis:**  **Indirect effects: Liraglutide:** ↑ delayed memory partially mediated by L hippocampus activation (bootstrapped; 2,000 samples); | **Liraglutide vs Baseline:** ↑ delayed memory, attention, executive function; ↓ BMI, body weight, body fat ratio, waist circumference;  **SEM Analysis:** direct model paths: ↓ body fat ratio → improved attention; ↑ fasting insulin → ↓ executive function test time  **Covariate adjustment:** continuous variables: baseline values;  **p-value correction:** bonferroni |
| Li et al., (2025)^24^ | Randomized parallel-group open-label | **T2DM: Liraglutide** (n=12; 52 years; BMI=27; F=6),  **Controls** (n=36; 54 years; BMI=23; F=19);  **Dapagliflozin** (n=12; 57 years; BMI=27; F=5), **Acarbose** (n=12; 56 years; BMI=27; F=5) | Liraglutide vs controls, dapagliflozin, oral acarbose; **Duration:** 16 weeks; **Dose:** 0.6-1.8 mg/day (increased to max 1.8 mg over 2 weeks) | fMRI: odor-visual task (lavender scent + paired visual; fresh air + paired visual; odor-induced Seed-Voxel FC); **Scan Timepoints:** baseline, 16 weeks; **Feeding State:** NR | Seed: Primary olfactory cortex (POC) | **Liraglutide vs Baseline:** NS effects  **All T2DM vs Controls - Baseline:** ↑ POC-L middle/superior frontal gyrus.  **Covariate adjustment:** age, sex, education  **p-value correction:** GRF | **Liraglutide vs Controls:** ↑ executive function, delayed memory, attention, olfactory threshold  **Liraglutide vs Baseline:** ↓ BMI, body weight, body fat, waist circumference, HbA1c |
| **fMRI: Memory and Learning Paradigms** | | | | | | | |
| **Author, Year** | **Study Type** | **Sample Description (Sample size; mean Age; mean BMI)** | **Intervention (GLP-1RA; Comparator; Duration; Dose)** | **Modality: Paradigm (Contrasts/Metric); Scan Timepoints; Scan Feeding State** | **Whole Brain/ROI** | **Imaging Outcomes** | **Behavioral/**  **Metabolic Outcomes** |
| Klausen et al., (2022)^17^ | Randomized double-blind placebo-controlled | **AUD: Exenatide** (n=28; 51 years; BMI=NR, F=12), **Placebo** (n=33; 49 years; BMI=NR; 13); **Controls** (n=25; 49 years; BMI=NR; F=10)  **fMRI Completers:** Exenatide=17, Placebo=16,  Controls=25 | Exenatide vs placebo, controls; **Duration:** 26 weeks; **Dose:** 2 mg/week | fMRI: N-Back Task (2-back vs 0-back; 2-back vs 1-back); **Scan Timepoints:** baseline, 26 weeks; **Feeding State:** NR | ROI: dPFC mask; Exploratory: Whole brain | **ROI Analysis:  Group x Timepoint Interaction:** **Baseline:** NS effects; **26 weeks:** ↓ R frontal pole, R superior frontal gyrus (2-back > 1-back)  **Whole Brain Analysis:** No additional significant differences  **Covariate adjustment:** NR  **p-value correction:** cluster-level | **Exenatide vs placebo – 26 weeks:** ↓ BMI, HbA1c; ↑ urinary oxidative stress markers; NS effects drinking days;  **BMI Subgroup Analysis:** BMI>30: Exenatide ↓ heavy drinking (−23.6%), ↓ total intake; BMI<25: Exenatide ↑ heavy drinking days (+27.5%).  **Craving:** Baseline: all-AUD vs Control: ↑ craving; 26-weeks: NS effects |
| Hanssen et al., (2023)^25^ | Randomized single-blind placebo-controlled crossover | **Insulin Sensitive-Lean (IS**+; n=18; 27 years; BMI=24; F=9), **Insulin Insensitive-Obese** (**IS-**; n=17; 27 years; BMI=33; F=11) | Liraglutide vs placebo; **Duration:** acute; **Dose:** 0.6 mg (evening before testing) | fMRI: Associative learning task; **Scan Timepoints:** placebo, liraglutide; **Feeding State:** fasted | Midbrain, striatum, vmPFC | **Group x Intervention Interaction:** **IS-:** ↑ adaptive prediction error encoding; **IS-:** ↑ encoding in vmPFC, dACC; **IS+:** NS effects;  **Covariate adjustment:** NR  **p-value correction:** FWE | **IS- vs IS+: Placebo:** IS-: ↓ learning rate, adaptive prediction error  **Liraglutide vs Placebo:** **IS-:** ↑ learning rate, adaptive prediction error; **IS+:** ↓ learning rate |

**Abbreviations:** ACC, anterior cingulate cortex; AUD, alcohol use disorder; BMI, body mass index; BP, blood pressure; CANTAB, Cambridge Neuropsychological Test Automated Battery; CNS, central nervous system; dPFC, dorsolateral prefrontal cortex; F, female; FDR, false discovery rate; fMRI, functional magnetic resonance imaging; FWE, family-wise error; HbA1c, glycated hemoglobin; HDL, high-density lipoprotein cholesterol; IS+, insulin sensitive; IS−, insulin insensitive; IV, intravenous; L, left; mg, milligram; NAc, nucleus accumbens; ng, nanogram; NR, not reported; NS, not significant; OFC, orbitofrontal cortex; PCOS, polycystic ovary syndrome; pmol/kg/min, picomoles per kilogram per minute; POC, primary olfactory cortex; R, right; SEM, standard error of the mean; SVC, small volume correction; T2DM, type 2 diabetes mellitus; µg, microgram; vmPFC, ventromedial prefrontal cortex; Δ, change; ↑, increase; ↓, decrease; +ve, positively; −ve, negatively.

**Table 2. fMRI: Resting-State Paradigm**

| **Author, Year** | **Study Type** | **Sample Description (Sample size; mean Age; mean BMI)** | **Intervention (GLP-1RA; Comparator; Duration; Dose)** | **Modality: Paradigm (Contrasts/Metric); Scan Timepoints; Scan Feeding State** | **Whole Brain/ROI** | **Imaging Outcomes** | **Behavioral/**  **Metabolic Outcomes** |
| --- | --- | --- | --- | --- | --- | --- | --- |
| Coveleskie et al., (2017)^26^ | Randomized double-blind placebo-controlled crossover | **Obese** (n=8; 25 years; BMI=34; F=8), **Lean** (n=11; 27 years; BMI=34; F=11) | Exenatide vs placebo; **Duration:** acute; **Dose:** 5 µg | rsfMRI: FC; **Scan Timepoints:** baseline, exenatide, placebo; **Feeding State:** fasted | Hypothalamus, thalamus, NTS | **Exenatide vs Placebo:** **Obese:** ↑ FC L NTS–L thalamus, L hypothalamus, ↑ FC R NTS–L thalamus**; Lean:** NS effects  **Covariate adjustment:** age, exenatide blood level  **Associations:** **Calorie intake: Post-Exenatide (All Subjects):** +ve correlated with R NTS–R hypothalamus, L thalamus; **Hunger**: **Baseline (Lean > Obese):** +ve correlated with R NTS–L thalamus; **Post-Exenatide (All Subjects):** +ve correlated R NTS–R hypothalamus; **Post-Exenatide (Obese > Lean):** +ve correlated with R NTS–L thalamus;  **Covariate adjustment (associations):** Exenatide blood level, age, drug day order  **p-value correction:** FDR | **Exenatide vs Placebo:** **Obese:** ↓ hunger; **NS effects:** ad libitum calorie consumption; satiety |
| Binda et al., (2019)^9^ | Within-subject controlled intervention, non-randomized | **Obese** (n=10; 45 years; BMI=33; F=5); **Lean** (n=10; 44 years; BMI=24; F=6) | Exenatide vs saline; **Duration:** acute; **Dose:** IV infusion, 0.3 µg loading dose, then 0.05 µg/min | rsfMRI: % raw signal intensity change; **Scan Timepoints:** saline, exenatide; **Feeding State:** fasted | Whole-cortical surface  ROIs: V1-V2, lateral occipital, fusiform gyrus, temporal pole | **Whole-cortical surface analysis:**  **Exenatide vs Saline (Drug Main Effect):** ↑ resting BOLD (widespread cortical regions);  **Obesity vs Lean (Group Main Effect):** ↓ resting BOLD (occipital, insula)  **ROI Analysis:**  **Exenatide vs Saline (Drug Main Effect):** ↑ BOLD (all ROIs);  **Obesity vs Lean (Group Main Effect):** ↓ BOLD (all ROIs but temporal pole);  **Associations: BMI (Exenatide > Saline):** -ve correlated with resting BOLD  **Covariate adjustment:** NR  **p-value correction:** Uncorrected | NR |
| Watson et al., (2019)^27^ | Randomized double-blind placebo-controlled | **Subjective cognitive complaints:** **Baseline:** **Liraglutide** (n=22; 61 years; BMI=29; F=14), **Placebo** (n=21; 62 years; BMI=30; F=11); **12-weeks:** **Liraglutide** (n=15; 62 years; F=11), **Placebo** (n=11, 60 years, F=8) | Liraglutide vs placebo; **Duration:** 12 weeks; **Dose:** 0.6-1.8 mg/day (weekly 0.6-mg escalation to max 1.8 mg) | rsfMRI: FC; **Scan Timepoints:** baseline, 12 weeks; **Feeding state**: NR | Seed: Hippocampus | **Liraglutide vs Placebo: Baseline:** NS Effects**; 12 weeks:** ↑ FC bilateral hippocampus-frontal cortex cluster, posterior cingulate/precuneus cluster, occipital/parietal cortex cluster  **Associations:** **Baseline:** ↓ FPG correlated with ↑ FC (bilateral hippocampus–L anterior cingulate/paracingulate, L inferior frontal gyrus, L caudate)  **Covariate adjustment:** age, sex  **p-value correction:** GRF | **Liraglutide vs Placebo:** **NS effects:** BMI, FPG, cognitive performance, cognitive complaints |
| Zhang et al., (2019)^22^ | Randomized parallel group intervention | **Obese-T2DM: Liraglutide** (n=9; 52 years; BMI=32 F=3),  **Exenatide** (n=10; 53 years; BMI=32; F=4); | Liraglutide vs exenatide; **Duration:** 3 months; **Dose:** liraglutide: 0.6-1.8 mg/day (increased to 1.8 mg over 2 weeks), exenatide: 5-10 µg twice daily (increased to max 10 µg over 2 weeks) | rsfMRI: FC; **Scan timepoints:** baseline, 3 months; **Feeding state:** NR | ‘Olfactory network’ Seed ROIs: Parahippocampus, amygdala, piriform cortex, insula, OFC, hippocampus, entorhinal cortices | **Liraglutide/Exenatide (3 months) vs Baseline:** NS effects;  **Exenatide vs Liraglutide:** NS effects  **Covariate adjustment:** age, sex, education, BP, serum lipids  **p-value correction:** GRF | **Liraglutide/Exenatide vs Baseline:** ↓ weight, BMI, HbA1c, systolic BP; ↑ 2-hour postprandial insulin, C-peptide, HDL  **Cognition/Olfaction - Liraglutide/Exenatide vs Baseline:** ↑ memory, general cognition, olfactory identification (**covariate adjustment:** age, sex, education, duration, BP); **With additional HbA1c/BMI adjustment:** NS effects;  **Liraglutide vs Exenatide: 3 months:** NS effects |
| Verovnik & Vovk, (2024)^28^ | Secondary analysis of randomized single-blind placebo-controlled | **PCOS: Semaglutide** (n=10; 31 years; Weight=99kg; F=10), **Placebo** (n=8; 34 years; Weight=95kg; F=8) | Semaglutide vs placebo; **Duration:** 16 weeks; **Dose:** 0.5-1.0 mg/week (increased to max 1.0 mg after 4 weeks) | rsfMRI: FC; **Scan Timepoints:** baseline, 16 weeks; **Feeding state:** NR | Amygdala, Insula, OFC, Precuneus, Parahippcampal gyrus, MTG, ACC | **Semaglutide vs Placebo – 16 weeks:** NS effects  **Within-Group (Baseline vs 16 weeks): Semaglutide:** ↑ FC in bilateral insula-bilateral amygdala, L parahippocampus-L amygdala; ↓ FC in L MTG-R MTG, R Precuneus-L insula; **Placebo:** ↓ FC in L MTG-bilateral precuneus, L precuneus-bilateral cingulate gyrus, R precuneus-bilateral cingulate gyrus; ↑ FC in L insula-L amygdala, R precuneus-R amygdala,  **Covariate adjustment:** NR  **p-value correction:** NR | **Semaglutide vs Placebo – 16 weeks:** ↓ Δ weight, waist circumference |
| Winzeler et al., (2021)^16^ | Exploratory fMRI substudy of randomized double-blind placebo-controlled crossover (with matched controls) | **Primary Polydipsia** (n=15; 32 years; BMI=NR, F=11), **Controls** (n=15; 29 years; BMI=NR; F=12) | Dulaglutide vs placebo, controls; **Duration:** 3 weeks; **Dose:** 1.5 mg/week | rsfMRI: Seed-Whole Brain FC; **Scan Timepoints:** baseline, 3-week endpoints (placebo, dulaglutide); **Feeding State:** fasted | ROI seeds: Hypothalamus, NAc, midbrain | **Dulaglutide vs Placebo:** NS effects  **Placebo vs Controls:** NS effects  **Covariate adjustment:** NR  **p-value correction:** cluster-level | **Dulaglutide vs Placebo**: ↓ fluid intake, urine output, thirst ratings; ↑ quality of life  **Covariate adjustment:** baseline measurement |
| Cheng et al., (2022)^23^ | Randomized parallel-group open-label | **T2DM: Liraglutide** (n=12; 52 years; BMI=27; F=6);  **Dapagliflozin** (n=12; 57 years; BMI=27; F=5), **Acarbose** (n=12; 56 years; BMI=27; F=5) | Liraglutide vs dapagliflozin, oral acarbose; **Duration:** 16 weeks; **Dose:** 0.6-1.8 mg/day (increased to max 1.8 mg over 2 weeks) | rsfMRI: Seed-whole brain FC, ALFF, ReHo; **Scan Timepoints:** baseline, 16 weeks; **Feeding State:** NR | ROI seeds: ‘Olfactory network’: Parahippocampus, amygdala, piriform cortex, insula, OFC, hippocampus, entorhinal cortices | **Liraglutide vs Baseline:** NS effects  **Covariate adjustment:** NR  **p-value correction:** GRF | **Liraglutide vs Baseline:** ↑ delayed memory, attention, executive function; ↓ BMI, body weight, body fat ratio, waist circumference;  **SEM Analysis:** direct model paths: ↓ body fat ratio → improved attention; ↑ fasting insulin → ↓ executive function test time  **Covariate adjustment:** continuous variables: baseline values  **p-value correction:** post-hoc bonferroni |

**Abbreviations:** ACC, anterior cingulate cortex; ALFF, amplitude of low-frequency fluctuations; BOLD, blood oxygen level–dependent; BP, blood pressure; F, female; FC, functional connectivity; FPG, fasting plasma glucose; GRF, Gaussian random field; HbA1c, glycated hemoglobin; HDL, high-density lipoprotein cholesterol; IV, intravenous; L, left; mg, milligram; MTG, middle temporal gyrus; NAc, nucleus accumbens; NR, not reported; NS, not significant; NTS, nucleus of the solitary tract; OFC, orbitofrontal cortex; PCOS, polycystic ovary syndrome; R, right; ReHo, regional homogeneity; ROI, region of interest; SEM, standard error of the mean; T2DM, type 2 diabetes mellitus; µg, microgram; V1, primary visual cortex; V2, secondary visual cortex; Δ, change; ↑, increase; ↓, decrease; +ve, positively; −ve, negatively.

**Table 3. Structural MRI**

| **Author, Year** | **Study Type** | **Sample Description (Sample size; mean Age; mean BMI)** | **Intervention (GLP-1RA; Comparator; Duration)** | **Modality: Paradigm (Contrasts/Metric); Scan Timepoints; Scan Feeding State** | **Whole Brain/ROI** | **Imaging Outcomes** | **Behavioral/**  **Metabolic Outcomes** |
| --- | --- | --- | --- | --- | --- | --- | --- |
| Mansur et al., (2017)^29^ | Secondary analysis of pilot, open-label study | **MDD or BD with impaired executive function** (n=17; 38 years; BMI=31; F=11) | Liraglutide vs baseline; **Duration:** 4 weeks; **Dose:** 0.6-1.8 mg/day (weekly 0.6-mg escalation to max 1.8 mg) | sMRI: GMV; **Scan Timepoints:** baseline, 4 weeks; **Feeding State:** fasted | Caudal middle frontal, rostral middle frontal, superior frontal, lateral OFC, medial OFC, hippocampus, amygdala, NAc, caudate, putamen, pallidum | **Liraglutide vs Baseline:** **Covariate unadjusted:** GMV ↑ L NAc; **Covariate adjusted (age, gender, ICV, BMI):** GMV ↑ L NAc, R putamen, R, pallidum, R rostral middle frontal, L lateral OFC; **Timepoint x BMI Interaction (adjusted for ICV):** R amygdala, L NAc, rostral middle frontal  **Associations (adjusted for ICV):** **Δ GMV–BMI:** ↓ BMI with ↑ GMV (bilateral caudate/putamen, frontal regions, R lateral OFC); ↓ BMI with ↓ GMV R NAc; **Δ GMV–Cognition:** ↓ TMT-B with ↑ GMV (L hippocampus, R amygdala); ↑ TMT-B with ↑ GMV (L caudate, R superior frontal, R lateral, medial OFC)  **p-value correction:** Uncorrected | **Liraglutide vs Baseline:** ↓ BMI, weight, fasting glucose, Hb1Ac, systolic BP |
| Mullins et al., (2019)^30^ | Randomized double-blind placebo-controlled | **Amnestic MCI or probable AD: Exenatide** (n=11; 72 years; BMI=27; F=4), **Placebo** (n=10; 74 years; BMI=27; F=6);  **sMRI Completers:** 6-month=21; 18-month=18 | Exenatide vs placebo; **Duration:** 18 months; **Dose:** 5-10 µg twice daily (increased to max 10 µg after 1 week) | sMRI: GM, CT; **Scan Timepoints:** baseline, 6 months, 12 months, 18 months; **Feeding State:** NR | Whole Brain | **Exenatide vs Placebo (****Group x Timepoint Interaction):** NS effects  **Timepoint (Main effect):** ↓ GMV in precuneus, medial temporal–parietal; ↓ CT  **Covariate adjustment:** age, sex, TIV; **p-value correction:** FWE/FDR | **Exenatide vs Placebo (Group x Timepoint Interaction):** ↑ Digit span (at 6-months only); ↓ glucose levels, GLP-1, CSF Aβ42  **Exenatide vs Baseline:** ↓ BMI (6-, 12-months only), glucose levels, GLP-1  **Timepoint (Main Effect):** ↓ MMSE, verbal fluency, digit-symbol total, CSF Aβ42; plasma Aβ40, |
| Li et al., (2025)^24^ | Randomized parallel-group open-label | **T2DM: Liraglutide** (n=12; 52 years; BMI=27; F=6), **Controls** (n=36; 54 years; BMI=23; F=19);  **Dapagliflozin** (n=12; 57 years; BMI=27; F=5), **Acarbose** (n=12; 56 years; BMI=27; F=5), | Liraglutide vs dapagliflozin, oral acarbose; **Duration:** 16 weeks; **Dose:** 0.6-1.8 mg/day (increased to max 1.8 mg over 2 weeks) | sMRI: TIV, GMV, WMV, CSFV, GMV/TIV; **Scan Timepoints:** baseline, 16 weeks; **Feeding State:** NR | Primary Olfactory Cortex | **Liraglutide vs Baseline:** NS effects;  **All-T2DM vs Controls:** NS effects;  **Covariate adjustment:** age, sex, education  **p-value correction:** FDR | **Liraglutide vs Baseline:** ↓ BMI, body weight, body fat, waist circumference, HbA1c  **Liraglutide vs Controls:** ↑ executive function, delayed memory, attention, olfactory threshold  **All-T2DM vs Controls:** ↑ BMI, body weight, body fat, waist circumference, HbA1c, BP, FPG, PPG, C-peptide, FSI; ↓ HDL, cognitive/executive function, olfactory scores; |
| Edison et al., (2026)^31^ | Randomized double-blind placebo-controlled | **Mild-moderate AD**: **Liraglutide** (n=102; 71 years; BMI=NR; F=42), **Placebo** (n=102; 73 years; BMI=NR; F=39)  **sMRI Completers:**  **Baseline:** Liraglutide=101, Placebo=101:  **Post-Treatment:** Liraglutide=75, Placebo=83 | Liraglutide vs placebo; **Duration:** 52 weeks; **Dose:** 0.6-1.8 mg/day (increased to max 1.8 mg within 4 weeks) | sMRI: Volume, VBM; **Scan Timepoints:** baseline, 52 weeks; **Feeding State:** NR | Ventricles, hippocampus, entorhinal cortex, medial temporal lobe, temporal lobe, cingulate, isthmus cingulate, frontal lobe, parietal lobe, occipital lobe, frontoparietal, whole GM, whole WM | **Liraglutide vs Placebo (Δ Baseline-52 weeks): Volume:** **↓** temporal, frontoparietal, parietal, total GM; **VBM:** slower **↓** hippocampus, frontal, temporal medial, parietal, temporal, whole GM, whole WM**;**  **Covariate adjustment:** baseline values, age, MMSE  **p-value correction:** Uncorrected | **Liraglutide vs Placebo (Δ Baseline-52 weeks):** slower **↓** in ADAS-cognitive and NTB-executive scores |

**Abbreviations:** ADAS-Cognitive, Alzheimer’s Disease Assessment Scale–Cognitive subscale; BMI, body mass index; BP, blood pressure; C-peptide, connecting peptide; CSF Aβ42, cerebrospinal fluid amyloid-β 42; CSFV, cerebrospinal fluid volume; CT, cortical thickness; F, female; FDR, false discovery rate; FPG, fasting plasma glucose; FSI, fasting serum insulin; FWE, family-wise error; GLP-1, glucagon-like peptide-1; GMV, gray matter volume; HDL, high-density lipoprotein cholesterol; ICV, intracranial volume; L, left; mg, milligram; MMSE, Mini-Mental State Examination; NAc, nucleus accumbens; NR, not reported; NS, not significant; NTB-executive, Neuropsychological Test Battery–Executive function; OFC, orbitofrontal cortex; POC, Primary Olfactory Cortex; plasma Aβ40, plasma amyloid-β 40; PPG, postprandial glucose; R, right; sMRI, structural magnetic resonance imaging; TMT-B, Trail Making Test-B; TIV, total intracranial volume; µg, microgram; VBM, voxel-based morphometry; WMV, white matter volume; Δ, change; ↑, increase; ↓, decrease; +ve, positively; −ve, negatively.

**Table 4. Molecular Imaging**

| **Author, Year** | **Study Type** | **Sample Description (Sample size; mean Age; mean BMI)** | **Intervention (GLP-1RA; Comparator; Duration)** | **Modality: Paradigm (Contrasts/Metric); Scan Timepoints; Scan Feeding State** | **Whole Brain/ROI** | **Imaging Outcomes** | **Behavioral/**  **Metabolic Outcomes** |
| --- | --- | --- | --- | --- | --- | --- | --- |
| Gejl et al., (2016)^32^ | Randomized  double-blind placebo-controlled | **Mild-AD: Liraglutide** (n=14; 63 years; BMI=25; F=8), **Placebo** (n=20; 67 years; BMI=25; F=5)  **PET Completers:** **PIB:** Liraglutide=13, Placebo=18;  **FDG:** Liraglutide=14, Placebo=17 | Liraglutide vs placebo; **Duration:** 26 weeks; **Dose:** 0.6-1.8 mg/day (weekly 0.6-mg escalation to max 1.8 mg) | PET: [^11^C]-PIB (PIB-BPnd; sCBF), [^18^F]-FDG (CMRglc); **Scan Timepoints:** baseline, 26 weeks; **Feeding State:** NR | Cingulate, precuneus, frontal, parietal, temporal, occipital lobes, cerebellum, cerebral cortex | **Within-Group (Baseline vs 26 weeks): Liraglutide:** PIB-BPnd: ↑ temporal, occipital; sCBF: NS effects; Δ CMRglc: NS effects; **Placebo:** PIB-BPnd: ↑ temporal; sCBF: ↑ frontal, parietal, occipital, cerebellum, global cortex; Δ CMRglc: ↓ precuneus, parietal, temporal, occipital, cerebellum**;**  **Covariate adjustment:** NR  **p-value correction:** Uncorrected | **Liraglutide vs Placebo:** ↓ FPG, BP, Δ BMI, Δ weight;  **NS effects:** cognitive scores, heart rate, HbA1c |
| Gejl et al., (2017)^33^ | Post-hoc analysis of randomized  double-blind placebo-controlled | **Mild-AD:** **Liraglutide** (n=14; 63 years; BMI=25; F=8), **Placebo** (n=19; 67 years; BMI=25; F=5); **Controls** (n=6; 63 years; BMI=NR; F=NR)  **PET Completers:** Liraglutide=14, Placebo=17, Control=6 | Liraglutide vs placebo; **Duration:** 26 weeks; **Dose:** 0.6-1.8 mg/day (weekly 0.6-mg escalation to max 1.8 mg) | PET: [^18^F]-FDG (Tmax, GEF, GUF); **Scan Timepoints:** baseline, 26 weeks; **Feeding State:** NR | Whole cortex | **Within-Group (Baseline vs 26 weeks): Liraglutide:** ↑ cortical Tmax; ↑ FDG uptake/clearance; ↑ glucose uptake/clearance; **Placebo:** NS effects;  **Liraglutide vs Placebo: Baseline:** ↓ Tmax; **26 weeks:** Tmax: NS effects  **Associations:** Tmax ↑ with disease duration; liraglutide normalized toward baseline  **Covariates adjustment:** NR  **p-value correction:** Tukey’s  **Associations: Baseline (pooled AD groups):** CMRglc ↓ with AD duration; CMRglc ↑ with total cognition score; GUF ↑ with cognition; CMRglc ↑ with Tmax; FDG net clearance ↑ with total cognitive score, ↓ with AD duration | See Gejl et al., 2016 |
| Edison et al., (2026)^31^ | Randomized double-blind placebo controlled | **Mild-moderate AD: Liraglutide** (n=102; 71 years; BMI=NR; F=42), **Placebo** (n=102; 73 years; BMI=NR; F=39;)  **PET Completers:**  **Baseline:** Liraglutide=93, Placebo=94:  **Post-Treatment:** Liraglutide=72, Placebo=82 | Liraglutide vs placebo; **Duration:** 52 weeks; **Dose:** 0.6-1.8 mg/day (increased to 1.8 mg within 4 weeks) | PET: [^18^F]-FDG (SUV); **Scan Timepoints:** Baseline, 52 weeks; **Feeding State:** NR | Hippocampus, medial temporal lobe, posterior cingulate | **Liraglutide vs Placebo (Δ Baseline-52 weeks):** NS effects  **Covariate adjustment:** baseline SUV, age, MMSE;  **p-value correction:** Uncorrected | **Liraglutide vs Placebo (Δ Baseline-52 weeks):** slower ↓ in ADAS-cognitive and NTB-executive scores |
| Jensen et al., (2020)^34^ | Open-label placebo-controlled repeated measures | **Healthy Controls** (n=10; 24 years; Weight=73kg; F=5) | Exenatide vs baseline (saline); **Duration:** acute; **Dose:** IV infusion, 0.2 pmol/kg/min for 30 min, then 0.1 pmol/kg/min | SPECT: [^123^I]-PE2I (DAT BPnd); **Scan Timepoints:** baseline, exenatide; **Feeding State:** Non-Fasted | Caudate, Putamen | **Exenatide vs Baseline:** NS effect DAT BPnd;  **Association:** NS correlation of DAT BPnd with plasma exenatide concentration  **Covariate adjustment:** NR  **p-value correction:** uncorrected | NR |
| Daniele et al., (2015)^35^ | Randomized double-blind placebo-controlled crossover | **Mild postprandial hyperglycemia** (n=15; 57 years; BMI=29; F=0) | Exenatide vs placebo; **Duration:** acute; **Dose:** 5 µg | PET: [^18^F]-FDG (CMRglc); **Scan Timepoints:** exenatide, placebo; **Feeding State:** Fed, OGTT during scan | **‘****Glucose homeostasis regulation’ ROI:** NTS, brainstem, insula, putamen, caudate, amygdala, limbic system, hypothalamus; **‘food reward system’ ROI:** orbitofrontal lobe, thalamus, cingulate, putamen, caudate, amygdala, limbic system | **Exenatide vs Placebo:** CMRglc during OGTT: ↑ total GM, total cortex (glucose homeostasis ROI, food reward ROI); ↓ hypothalamus  **Association:** Glucose RaO, RaT -ve correlated with ROIs, total GM, NTS, insula, limbic system, thalamus, ACC, PCC, orbitofrontal, occipital, parietal, temporal after exenatide only;  **Covariate adjustment:** NR  **p-value correction:** Uncorrected | **Exenatide:** NS effects: FPG, FPI |
| Athauda et al., (2017)^36^ | Randomized double-blind placebo-controlled | **PD: Exenatide** (n=31; 62 years; BMI=NR; F=9), **Placebo** (n=29; 58 years; BMI=NR; F=7) | Exenatide vs placebo; **Duration:** 48 weeks; **Dose:** 2 mg/week | SPECT: DaTSCAN [¹²³I]-FP-CIT (DAT availability); **Scan Timepoints:** baseline, 60 weeks (12 weeks post-treatment withdrawal); **Feeding State:** NR | Caudate, Putamen | **Within-Group (Baseline vs 60 weeks): Exenatide:** ↓ DAT availability in bilateral caudate; **Placebo:** ↓ DAT availability in bilateral caudate, L putamen;  **Exenatide vs Placebo (**Δ **over time):** slower ↓ DAT availability in bilateral putamen, R caudate  **Covariate adjustment:** baseline Δ DAT availability, PD severity (Hoehn and Yahr), Levodopa equivalent dose  **p-value correction:** Uncorrected | **Exenatide vs Placebo:** Δ ↓ MDS-UPDRS – part 3 (off medication; 48 and 60 weeks);  **NS effects:** cognition, mood, dyskinesia, non-motor symptoms, quality of life, weight loss (> in exenatide), nausea, loss of appetite, abdominal pain; |
| Klausen et al., (2022)^17^ | Substudy within randomized double-blind placebo-controlled | **Baseline Sample**: **AUD-Exenatide** (n=20; 52 years; BMI=NR, F=9), **AUD-Placebo** (n=25; 55 years; BMI=NR; 7; n=25)  **Controls** (49 years; BMI=NR; F=10; PET-sample=21)  **Longitudinal PET Sample:** **Baseline:** Exenatide=7, Placebo=9;  **Post-Treatment**: Exenatide=7, Placebo=9; | Exenatide vs placebo, Controls; **Duration:** 26 weeks; **Dose:** 2 mg/week | SPECT: [^123^I]-PE2I (DAT BPnd); **Scan Timepoints:** baseline, 26 weeks; **Feeding State:** NR | Whole striatum, caudate, putamen | **All-AUD vs Controls (Baseline Sample):** NS effects; **Covariate adjustment:** baseline DAT BPnd  **Exenatide vs Placebo (26 weeks; Longitudinal PET Sample):** DAT BPnd: ↓ striatum, caudate, putamen; **Covariate adjustment:** age  **p-value correction:** uncorrected | **Exenatide vs placebo – 26 weeks:** ↓ BMI, HbA1c; ↑ urinary oxidative stress markers; **NS effect** drinking days  **BMI Subgroup Analysis:** BMI>30: Exenatide ↓ heavy drinking (−23.6%), ↓ total intake; BMI<25: Exenatide ↑ heavy drinking days (+27.5%).  **Craving** **(all-AUD vs Control): Baseline:** ↑ craving; **26 weeks**: NS effects |

**Abbreviations:** AD, Alzheimer’s disease; ADAS, Alzheimer’s Disease Assessment Scale; AUD, Alcohol Use Disorder; BMI, body mass index; BPnd, non-displaceable binding potential; CMRglc, cerebral glucose metabolic rate; DAT, dopamine transporter; F, female; FDG, [¹⁸F]-fluorodeoxyglucose; GEF, glucose extraction fraction; GUF, glucose uptake fraction; HbA1c, glycated hemoglobin; IV, intravenous; kg, kilograms; L, left; MDS-UPDRS, Movement Disorder Society–Unified Parkinson’s Disease Rating Scale; MMSE, Mini-Mental State Examination; NR, not reported; NS, not significant; NTB, neuropsychological test battery; OGTT, Oral Glucose Tolerance Test; PD, Parkinson’s disease; PET, positron emission tomography; pmol/kg/min, picomoles per kilogram per minute; RaO, rate of glucose absorption; RaT, total rate of glucose appearance; R, right; ROI, region of interest; sCBF, cerebral blood flow; SPECT, single-photon emission computed tomography; SUV, standard uptake value; Tmax, blood–brain glucose transport capacity; µg, microgram; Δ, change; ↑, increase; ↓, decrease; +ve, positively; −ve, negatively.

**Table 5: iEEG**

| **Author, Year** | **Study Type** | **Sample Description (Sample size; mean Age; mean BMI)** | **Intervention (GLP-1RA; Comparator; Duration)** | **Modality: Paradigm (Contrasts/Metric); Scan Timepoints; Scan Feeding State** | **Whole Brain/ROI** | **Imaging Outcomes** | **Behavioral/**  **Metabolic Outcomes** |
| --- | --- | --- | --- | --- | --- | --- | --- |
| Choi et al., (2025)^37^ | Single uncontrolled case-study within randomized placebo-controlled | **Persistent Obesity+Eating LOC/food preoccupation post-bariatric surgery** (n=3; 57 years; BMI=47; F=3); **Tirzepatide**=1, **Controls**=2 | Tirzepatide vs controls; **Tirzepatide duration/dose:** 11 months; 2.5–7.5 mg/week pre-implantation (4 months); escalated to 12.5 mg/week during post-implantation months 1–4, then 15 mg/week during months 4–7 | Intracranial EEG (rDBS during severe food preoccupation, number of food preoccupation sessions); **Scan Timepoints:** NA; **Feeding State:** NA | NAc | **Controls:** **Biomarker discovery phase:** ↑ NAc delta-theta band (preoccupation vs control states); **Stimulation phase:** NS effects (preoccupation vs control states), ↓ food preoccupation episodes  **Tirzepatide: Biomarker discovery phase (Months 2-7):** **Months 2–4 (dose escalation):** NS difference (preoccupation vs control states; no distinct biomarker), ↓ preoccupation episodes; **Months 5–7 (max dose):** ↑ bilateral NAc delta–theta band (preoccupation vs control; biomarker re-emergence), ↑ preoccupation episodes  **Covariate adjustment:** NR  **p-value correction:** permutation-testing (two-sided, 1,000-10,000 permutations; p<0.05) with cluster correction | **Tirzepatide: Pre-implant:** n=19 food preoccupation episodes; **Post-implant:** **Months 2-4:** ↓ preoccupation episodes (n=5), ↓ body weight (7%); **Months 5-7:** ↑ preoccupation episodes (n=11) |

**Abbreviations:** BMI, body mass index; F, Female; LOC, loss of control; mg, milligram; NA, not applicable; NS, not significant; ↑, increase; ↓, decrease.

**Table 6.** Risk of bias/quality assessment of the included studies using the NIH Quality Assessment Tool of Controlled Intervention Studies

| **Study** | **Item** | | | | | | | | | | | | | | | | **Quality Rating** |
| --- | --- | --- | --- | --- | --- | --- | --- | --- | --- | --- | --- | --- | --- | --- | --- | --- | --- |
|  | **1** | **2** | **3** | **4** | **5** | **6** | **7** | **8** | **9** | **10** | **11** | **12** | **13** | **14** | **15^a^** | **16^b^** |  |
| Schlögl et al., (2013)^1^ | ✓ | NR | NR | ✓ | X | ✓ | ✓ | NA | NA | ✓ | ✓ | X | ✓ | ✓ | ✓ | NR | Good |
| van Bloemendaal et al., (2014)^2^ | ✓ | NR | NR | X | X | ✓ | ✓ | ✓ | NA | X | ✓ | X | ✓ | ✓ | ✓ | NR | Fair |
| Daniele et al., (2015)^35^ | ✓ | NR | NR | ✓ | X | NA | ✓ | ✓ | ✓ | ✓ | ✓ | X | NA | ✓ | X | NR | Fair |
| ten Kulve et al., (2015)^3^ | ✓ | NR | X | X | X | ✓ | ✓ | ✓ | NR | ✓ | ✓ | X | ✓ | ✓ | ✓ | ✓ | Good |
| van Bloemendaal et al., (2015)^18^ | ✓ | NR | NR | NR | NR | ✓ | ✓ | ✓ | NA | ✓ | ✓ | X | ✓ | ✓ | ✓ | NR | Good |
| van Bloemendaal et al., (2015)^4^ | ✓ | NR | NR | NR | NR | ✓ | ✓ | ✓ | NA | ✓ | ✓ | X | ✓ | ✓ | ✓ | ✓ | Good |
| Eldor et al., (2016)^5^ | X | NA | NA | NA | X | ✓ | ✓ | ✓ | NA | ✓ | ✓ | X | ✓ | NA | ✓ | NR | Fair |
| Farr et al., (2016)^6^ | ✓ | NR | NR | ✓ | NR | ✓ | CD | CD | NR | ✓ | ✓ | X | ✓ | ✓ | ✓* | ✓ | Good |
| Farr et al., (2016)^7^ | ✓ | NR | NR | ✓ | NR | ✓ | ✓ | ✓ | NR | ✓ | ✓ | X | ✓ | ✓ | X | NR | Good |
| Gejl et al., (2016)^32^ | ✓ | ✓ | ✓ | ✓ | ✓ | ✓ | ✓ | X | NR | ✓ | ✓ | ✓ | ✓ | ✓ | X | NR | Good |
| ten Kulve et al., (2016)^19^ | ✓ | NR | X | X | NR | ✓ | ✓ | ✓ | NR | ✓ | ✓ | X | ✓ | ✓ | ✓ | NR | Good |
| Athauda et al., (2017)^36^ | ✓ | ✓ | NR | ✓ | X | CD | ✓ | ✓ | ✓ | ✓ | ✓ | X | ✓ | ✓ | X | ✓ | Good |
| Coveleskie et al., (2017)^26^ | ✓ | NR | ✓ | ✓ | NR | ✓ | ✓ | ✓ | NA | ✓ | ✓ | X | ✓ | ✓ | ✓ | ✓ | Good |
| Gejl et al., (2017)^33^ | ✓ | NR | ✓ | ✓ | ✓ | X | ✓ | X | NR | NR | ✓ | CD | ✓ | ✓ | ✓ | NR | Good |
| Bae et al., (2019)^8^ | ✓ | NR | CD | X | CD | ✓ | ✓ | NA | NA | ✓ | ✓ | ✓ | ✓ | ✓ | X | NR | Fair |
| Binda et al., (2019)^9^ | X | NA | NA | X | CD | ✓ | ✓ | ✓ | NA | ✓ | ✓ | X | ✓ | ✓ | X | NR | Fair |
| Farr et al., (2019)^10^ | ✓ | NR | NR | ✓ | NR | NA | ✓ | NR | NR | ✓ | ✓ | ✓ | ✓ | ✓ | ✓ | ✓ | Good |
| Mullins et al., (2019)^30^ | ✓ | NR | CD | ✓ | X | ✓ | X | NR | NR | ✓ | ✓ | X | ✓ | ✓ | ✓ | ✓ | Good |
| Watson et al., (2019)^27^ | ✓ | ✓ | CD | ✓ | NR | ✓ | ✓ | ✓ | CD | ✓ | ✓ | X | ✓ | ✓ | ✓ | ✓ | Good |
| Jensen et al., (2020)^34^ | X | NA | NA | NA | CD | NA | ✓ | ✓ | NA | NR | ✓ | X | ✓ | NA | X | X | Fair |
| Winzeler et al., (2021)^16^ | ✓ | ✓ | ✓ | ✓ | NR | ✓ | ✓ | ✓ | ✓ | ✓ | ✓ | X | ✓ | ✓ | ✓ | NR | Good |
| Cheng et al., (2022)^23^ | ✓ | ✓ | X | X | X | ✓ | ✓ | X | NR | ✓ | ✓ | ✓ | ✓ | ✓ | ✓ | NR | Good |
| Klausen et al., (2022)^17^ | ✓ | ✓ | ✓ | ✓ | NR | CD | ✓ | ✓ | ✓ | ✓ | ✓ | X | ✓ | ✓ | ✓** | ✓ | Good |
| van Ruiten et al., (2022)^11^ | ✓ | ✓ | ✓ | ✓ | NR | CD | ✓ | ✓ | NR | ✓ | ✓ | X | ✓ | ✓ | ✓ | NR | Good |
| van Ruiten et al., (2022)^12^ | ✓ | CD | X | X | NR | ✓ | ✓ | ✓ | NR | ✓ | ✓ | ✓ | ✓ | ✓ | ✓ | NR | Good |
| van Ruiten et al., (2022)^20^ | ✓ | ✓ | ✓ | ✓ | NR | CD | ✓ | ✓ | NR | ✓ | ✓ | ✓ | ✓ | ✓ | ✓ | NR | Good |
| van Ruiten et al., (2022)^13^ | ✓ | ✓ | ✓ | ✓ | NR | CD | ✓ | ✓ | NR | ✓ | ✓ | ✓ | ✓ | ✓ | ✓ | NR | Good |
| Coppin et al., (2023)^21^ | ✓ | NR | ✓ | ✓ | NR | ✓ | ✓ | CD | NR | ✓ | ✓ | X | ✓ | ✓ | ✓ | NR | Good |
| Hanssen et al., (2023)^25^ | ✓ | NR | CD | X | CD | ✓ | ✓ | ✓ | NA | ✓ | ✓ | CD | ✓ | ✓ | ✓ | NR | Good |
| Verovnik & Vovk, (2024)^28^ | ✓ | CD | CD | CD | CD | ✓ | ✓ | CD | NR | CD | ✓ | X | ✓ | ✓ | X | NR | Fair |
| Jensterle et al., (2025)^14^ | ✓ | NR | CD | X | CD | ✓ | ✓ | ✓ | NR | ✓ | ✓ | X | ✓ | ✓ | ✓ | NR | Good |
| Li et al., (2025)^24^ | ✓ | ✓ | X | X | X | ✓ | ✓ | ✓ | NR | ✓ | ✓ | ✓ | ✓ | ✓ | ✓ | ✓ | Good |
| Martin et al., (2025)^15^ | ✓ | ✓ | X | X | X | CD | ✓ | X | NR | ✓ | ✓ | X | ✓ | ✓ | X | ✓ | Fair |
| Edison et al., (2026)^31^ | ✓ | ✓ | NR | ✓ | NR | ✓ | ✓ | ✓ | ✓ | ✓ | ✓ | X | ✓ | ✓ | X | ✓ | Good |

Items 1-14 are those from the NIH Quality Assessment Tool of Controlled Intervention Studies. Items 15 and 16 are neuroimaging-specific items we additionally included. Abbreviations: a = Controlled for multiple comparisons; b = utilized/reported covariates in statistical analysis; CD = cannot determine; NA = Not applicable; NR = Not reported; Symbols: ✓ = Yes; X = No; * = Multiple comparisons applied only for ROI analysis; ** = Multiple comparisons applied only for whole brain analysis

**Table 7.** Risk of bias/quality assessment of the included studies using the NIH Quality Assessment Tool of Pre-Post Studies

| **Study** | **Item** | | | | | | | | | | | | | | **Quality Rating** |
| --- | --- | --- | --- | --- | --- | --- | --- | --- | --- | --- | --- | --- | --- | --- | --- |
|  | **1** | **2** | **3** | **4** | **5** | **6** | **7** | **8** | **9** | **10** | **11** | **12** | **13^a^** | **14^b^** |  |
| Mansur et al., (2017)^29^ | ✓ | ✓ | ✓ | CD | X | ✓ | ✓ | X | ✓ | ✓ | ✓ | ✓ | ✓ | ✓ | Good |
| Zhang et al., (2019)^22^ | ✓ | ✓ | ✓ | NR | X | ✓ | ✓ | X | ✓ | ✓ | ✓ | ✓ | X | ✓ | Good |

Items 1-12 are those from the NIH Quality Assessment Tool of Pre-Post Studies. Items 13 and 14 are neuroimaging-specific items we additionally included. Abbreviations: a = Controlled for multiple comparisons; b = utilized covariates in statistical analysis; CD = cannot determine; NA = Not applicable; NR = Not reported; Symbols: ✓ = Yes; X = No;

**Table 8.** Risk of bias/quality assessment of the included studies using the NIH Quality Assessment Tool of Case Series

| **Study** | **Item** | | | | | | | | | | | | | | | | **Quality Rating** |
| --- | --- | --- | --- | --- | --- | --- | --- | --- | --- | --- | --- | --- | --- | --- | --- | --- | --- |
|  | **1** | **2** | **3** | **4** | **5** | **6** | **7** | **8** | **9** | **10** | **11** | **12** | **13** | **14** | **15^a^** | **16^b^** |  |
| Choi et al., (2025)^37^ | ✓ | ✓ | NR | ✓ | ✓ | ✓ | ✓ | **X** | **X** | ✓ | ✓ | **X** | ✓ | **X** | ✓ | NR | Good |

Items 1-14 are those from the NIH Quality Assessment Tool of Case Series. Items 15 and 16 are neuroimaging-specific items we additionally included. Abbreviations: a = Controlled for multiple comparisons; b = utilized covariates in statistical analysis; CD = cannot determine; NA = Not applicable; NR = Not reported; Symbols: ✓ = Yes; X = No;

**References**

1.	Schlögl H, Kabisch S, Horstmann A, et al. Exenatide-Induced Reduction in Energy Intake Is Associated With Increase in Hypothalamic Connectivity. *Diabetes Care*. 2013;36(7):1933-1940. doi:10.2337/dc12-1925

2.	van Bloemendaal L, IJzerman RG, ten Kulve JS, et al. GLP-1 Receptor Activation Modulates Appetite- and Reward-Related Brain Areas in Humans. *Diabetes*. 2014;63(12):4186-4196. doi:10.2337/db14-0849

3.	ten Kulve JS, Veltman DJ, van Bloemendaal L, et al. Liraglutide Reduces CNS Activation in Response to Visual Food Cues Only After Short-term Treatment in Patients With Type 2 Diabetes. *Diabetes Care*. 2015;39(2):214-221. doi:10.2337/dc15-0772

4.	van Bloemendaal L, Veltman DJ, ten Kulve JS, et al. Emotional eating is associated with increased brain responses to food-cues and reduced sensitivity to GLP-1 receptor activation. *Obesity*. 2015;23(10):2075-2082. doi:10.1002/oby.21200

5.	Eldor R, Daniele G, Huerta C, et al. Discordance Between Central (Brain) and Pancreatic Action of Exenatide in Lean and Obese Subjects. *Diabetes Care*. 2016;39(10):1804-1810. doi:10.2337/dc15-2706

6.	Farr OM, Tsoukas MA, Triantafyllou G, et al. Short-term administration of the GLP-1 analog liraglutide decreases circulating leptin and increases GIP levels and these changes are associated with alterations in CNS responses to food cues: A randomized, placebo-controlled, crossover study. *Metabolism*. 2016;65(7):945-953. doi:10.1016/j.metabol.2016.03.009

7.	Farr OM, Sofopoulos M, Tsoukas MA, et al. GLP-1 receptors exist in the parietal cortex, hypothalamus and medulla of human brains and the GLP-1 analogue liraglutide alters brain activity related to highly desirable food cues in individuals with diabetes: a crossover, randomised, placebo-controlled trial. *Diabetologia*. 2016;59(5):954-965. doi:10.1007/s00125-016-3874-y

8.	Bae JH, Choi HJ, Cho KIK, Kim LK, Kwon JS, Cho YM. Glucagon-Like Peptide-1 Receptor Agonist Differentially Affects Brain Activation in Response to Visual Food Cues in Lean and Obese Individuals with Type 2 Diabetes Mellitus. *Diabetes Metab J*. 2019;44(2):248-259. doi:10.4093/dmj.2019.0018

9.	Binda P, Eldor R, Huerta C, et al. Exenatide modulates visual cortex responses. *Diabetes Metab Res Rev*. 2019;35(6):e3167. doi:10.1002/dmrr.3167

10.	Farr OM, Upadhyay J, Rutagengwa C, et al. Longer-term liraglutide administration at the highest dose approved for obesity increases reward-related orbitofrontal cortex activation in response to food cues: Implications for plateauing weight loss in response to anti-obesity therapies. *Diabetes Obes Metab*. 2019;21(11):2459-2464. doi:10.1111/dom.13827

11.	van Ruiten CC, Veltman DJ, Nieuwdorp M, IJzerman RG. Brain Activation in Response to Low-Calorie Food Pictures: An Explorative Analysis of a Randomized Trial With Dapagliflozin and Exenatide. *Front Endocrinol*. 2022;13. doi:10.3389/fendo.2022.863592

12.	van Ruiten CC, ten Kulve JS, van Bloemendaal L, Nieuwdorp M, Veltman DJ, IJzerman RG. Eating behavior modulates the sensitivity to the central effects of GLP-1 receptor agonist treatment: a secondary analysis of a randomized trial. *Psychoneuroendocrinology*. 2022;137:105667. doi:10.1016/j.psyneuen.2022.105667

13.	van Ruiten CC, Veltman DJ, Schrantee A, et al. Effects of Dapagliflozin and Combination Therapy With Exenatide on Food-Cue Induced Brain Activation in Patients With Type 2 Diabetes. *J Clin Endocrinol Metab*. 2022;107(6):e2590-e2599. doi:10.1210/clinem/dgac043

14.	Jensterle M, Kovac J, Vovk A, et al. Semaglutide and Taste in Women With Obesity and Polycystic Ovary Syndrome: A Randomized Placebo-Controlled Study. *J Clin Endocrinol Metab*. Published online May 9, 2025:dgaf278. doi:10.1210/clinem/dgaf278

15.	Martin CK, Carmichael OT, Carnell S, et al. Tirzepatide on ingestive behavior in adults with overweight or obesity: a randomized 6-week phase 1 trial. *Nat Med*. 2025;31(9):3141-3150. doi:10.1038/s41591-025-03774-9

16.	Winzeler B, Sailer CO, Coynel D, et al. A randomized controlled trial of the GLP-1 receptor agonist dulaglutide in primary polydipsia. *J Clin Invest*. 2021;131(20). doi:10.1172/JCI151800

17.	Klausen MK, Jensen ME, Møller M, et al. Exenatide once weekly for alcohol use disorder investigated in a randomized, placebo-controlled clinical trial. *JCI Insight*. 2022;7(19):e159863. doi:10.1172/jci.insight.159863

18.	van Bloemendaal L, Veltman DJ, ten Kulve JS, et al. Brain reward-system activation in response to anticipation and consumption of palatable food is altered by glucagon-like peptide-1 receptor activation in humans. *Diabetes Obes Metab*. 2015;17(9):878-886. doi:10.1111/dom.12506

19.	ten Kulve JS ten, Veltman DJ, Bloemendaal L van, et al. Endogenous GLP1 and GLP1 analogue alter CNS responses to palatable food consumption. Published online April 1, 2016. doi:10.1530/JOE-15-0461

20.	van Ruiten CC, Veltman DJ, Wijdeveld M, et al. Combination therapy with exenatide decreases the dapagliflozin-induced changes in brain responses to anticipation and consumption of palatable food in patients with type 2 diabetes: A randomized controlled trial. *Diabetes Obes Metab*. 2022;24(8):1588-1597. doi:10.1111/dom.14732

21.	Coppin G, Muñoz Tord D, Pool ER, et al. A randomized controlled trial investigating the effect of liraglutide on self-reported liking and neural responses to food stimuli in participants with obesity. *Int J Obes*. 2023;47(12):1224-1231. doi:10.1038/s41366-023-01370-w

22.	Zhang Z, Zhang B, Wang X, et al. Olfactory Dysfunction Mediates Adiposity in Cognitive Impairment of Type 2 Diabetes: Insights From Clinical and Functional Neuroimaging Studies. *Diabetes Care*. 2019;42(7):1274-1283. doi:10.2337/dc18-2584

23.	Cheng H, Zhang Z, Zhang B, et al. Enhancement of Impaired Olfactory Neural Activation and Cognitive Capacity by Liraglutide, but Not Dapagliflozin or Acarbose, in Patients With Type 2 Diabetes: A 16-Week Randomized Parallel Comparative Study. *Diabetes Care*. 2022;45(5):1201-1210. doi:10.2337/dc21-2064

24.	Li X, Zhang Y, Bi Y, et al. Dapagliflozin restores odour‐induced functional integration of primary olfactory cortex circuit but not olfactory‐related regional brain activation in patients with type 2 diabetes: A 16‐week randomised comparative study. *Diabetes Obes Metab*. 2025;27(12):7309-7322. doi:10.1111/dom.70132

25.	Hanssen R, Rigoux L, Kuzmanovic B, et al. Liraglutide restores impaired associative learning in individuals with obesity. *Nat Metab*. 2023;5(8):1352-1363. doi:10.1038/s42255-023-00859-y

26.	Coveleskie K, Kilpatrick LA, Gupta A, et al. The effect of the GLP‐1 analogue Exenatide on functional connectivity within an NTS‐based network in women with and without obesity. *Obes Sci Pract*. 2017;3(4):434-445. doi:10.1002/osp4.124

27.	Watson KT, Wroolie TE, Tong G, et al. Neural correlates of liraglutide effects in persons at risk for Alzheimer’s disease. *Behav Brain Res*. 2019;356:271-278. doi:10.1016/j.bbr.2018.08.006

28.	Verovnik B, Vovk A. Semaglutide, suicidal ideation and behaviour: A resting state functional magnetic resonance imaging perspective. *Diabetes Obes Metab*. 2024;26(2):782-784. doi:10.1111/dom.15363

29.	Mansur RB, Zugman A, Ahmed J, et al. Treatment with a GLP−1R agonist over four weeks promotes weight loss-moderated changes in frontal-striatal brain structures in individuals with mood disorders. *Eur Neuropsychopharmacol*. 2017;27(11):1153-1162. doi:10.1016/j.euroneuro.2017.08.433

30.	Mullins RJ, Mustapic M, Chia CW, et al. A Pilot Study of Exenatide Actions in Alzheimer’s Disease. *Curr Alzheimer Res*. 2019;16(8):741-752. doi:10.2174/1567205016666190913155950

31.	Edison P, Femminella GD, Ritchie C, et al. Liraglutide in mild to moderate Alzheimer’s disease: a phase 2b clinical trial. *Nat Med*. 2026;32(1):353-361. doi:10.1038/s41591-025-04106-7

32.	Gejl M, Gjedde A, Egefjord L, et al. In Alzheimer’s Disease, 6-Month Treatment with GLP-1 Analog Prevents Decline of Brain Glucose Metabolism: Randomized, Placebo-Controlled, Double-Blind Clinical Trial. *Front Aging Neurosci*. 2016;8:108. doi:10.3389/fnagi.2016.00108

33.	Gejl M, Brock B, Egefjord L, Vang K, Rungby J, Gjedde A. Blood-Brain Glucose Transfer in Alzheimer’s disease: Effect of GLP-1 Analog Treatment. *Sci Rep*. 2017;7:17490. doi:10.1038/s41598-017-17718-y

34.	Jensen ME, Galli A, Thomsen M, et al. Glucagon-like peptide-1 receptor regulation of basal dopamine transporter activity is species-dependent. *Neurochem Int*. 2020;138:104772. doi:10.1016/j.neuint.2020.104772

35.	Daniele G, Iozzo P, Molina-Carrion M, et al. Exenatide Regulates Cerebral Glucose Metabolism in Brain Areas Associated With Glucose Homeostasis and Reward System. *Diabetes*. 2015;64(10):3406-3412. doi:10.2337/db14-1718

36.	Athauda D, Maclagan K, Skene SS, et al. Exenatide once weekly versus placebo in Parkinson’s disease: a randomised, double-blind, placebo-controlled trial. *The Lancet*. 2017;390(10103):1664-1675. doi:10.1016/S0140-6736(17)31585-4

37.	Choi W, Nho YH, Qiu L, et al. Brain activity associated with breakthrough food preoccupation in an individual on tirzepatide. *Nat Med*. Published online November 17, 2025:1-6. doi:10.1038/s41591-025-04035-5
